# Acceptability and appropriateness of a novel parent-staff co-leadership model for childhood obesity prevention in Head Start: a qualitative interview study

**DOI:** 10.1186/s12889-021-10159-3

**Published:** 2021-01-22

**Authors:** Jacob P. Beckerman-Hsu, Cristina Gago, Alyssa Aftosmes-Tobio, Janine M. Jurkowski, Kindra Lansburg, Jessie Leonard, Merieka Torrico, Sebastien Haneuse, S. V. Subramanian, Erica L. Kenney, Kirsten K. Davison

**Affiliations:** 1 Boston College School of Social Work, McGuinn Hall 106K, 140 Commonwealth Ave, Chestnut Hill, MA 02467 USA; 2 Department of Nutrition, Harvard T.H. Chan School of Public Health, 665 Huntington Ave, Boston, MA 02115 USA; 3 Department of Health Policy, Management, & Behavior, University at Albany School of Public Health, 1 University Pl, Rensselaer, NY 121440 USA; 4 Action for Boston Community Development, 178 Tremont Street, Boston, MA 02111 USA; 5 Community Action Agency of Somerville, 66 Union Square, Somerville, MA 02143 USA; 6 Department of Biostatistics, Harvard T.H. Chan School of Public Health, 665 Huntington Ave, Boston, MA 02115 USA; 7 Department of Social and Behavioral Sciences, Harvard T.H. Chan School of Public Health, 677 Huntington Ave, Boston, MA 02115 USA; 8 Harvard Center for Population & Development Studies, 9 Bow Street, Cambridge, MA 02138 USA; 9 Department of Nutrition and Department of Social and Behavioral Sciences, Harvard T.H. Chan School of Public Health, 665 Huntington Ave, Boston, MA 02115 USA

**Keywords:** Peer leadership, Implementation outcomes, Parents, Obesity prevention, Early childhood education, Head Start

## Abstract

**Background:**

Peer leadership can be an effective strategy for implementing health programs, benefiting both program participants and peer leaders. To realize such benefits, the peer leader role must be appropriate for the community context. Also, peer leaders must find their role acceptable (i.e., satisfactory) to ensure their successful recruitment and retention. To date, parent peer leaders have seldom been part of early childhood obesity prevention efforts. Moreover, parents at Head Start preschools have rarely been engaged as peer leaders. The aim of this study is to evaluate the appropriateness and acceptability of an innovative model for engaging parents as peer leaders for this novel content area (early childhood obesity prevention) and setting (Head Start).

**Methods:**

Parents Connect for Healthy Living (PConnect) is a 10-session parent program being implemented in Head Start preschools as part of the Communities for Healthy Living early childhood obesity prevention trial. PConnect is co-led by a parent peer facilitator who is paired with a Head Start staff facilitator. In the spring of 2019, 10 PConnect facilitators participated in a semi-structured interview about their experience. Interview transcripts were analyzed by two coders using an inductive-deductive hybrid analysis. Themes were identified and member-checked with two interviewees.

**Results:**

Themes identified applied equally to parent and staff facilitators. Acceptability was high because PConnect facilitators were able to learn and teach, establish meaningful relationships, and positively impact the parents participating in their groups, although facilitators did express frustration when low attendance limited their reach. Appropriateness was also high, as PConnect provided adequate structure and support without being overly rigid, and facilitators were able to overcome most challenges they encountered.

**Conclusions:**

The PConnect co-facilitation model was highly acceptable and appropriate for both the parent facilitators (peer leaders) and the staff facilitators. Including parents as peer leaders aligns to Head Start’s emphasis on parent engagement, making it a strong candidate for sustained implementation in Head Start. The insights gained about the drivers of peer leadership appropriateness and acceptability in this particular context may be used to inform the design and implementation of peer-led health programs elsewhere.

**Trial registration:**

clinicaltrials.gov, NCT03334669 (7–11-17).

**Supplementary Information:**

The online version contains supplementary material available at 10.1186/s12889-021-10159-3.

## Background

### Peer leadership in health promotion

Peer leadership is an effective strategy for health promotion [1–7]. Peers, who are not professionals in health fields, take on a variety of leadership roles in health promotion programs [6]. While their titles vary (e.g., “community health worker,” “promotores,” “peer supporter”), their key functions include practical assistance and emotional support in utilizing healthcare and other resources, as well as managing complex health behaviors like medication adherence and healthy diet [6]. Compared to non-peers, peers can be more successful in establishing trust with community members, providing culturally appropriate services, and serving otherwise seldom-reached populations [8, 9]. Another unique benefit of peer leadership is the impact it has on peer leaders, who build skills and knowledge they use to improve their own health and the health of their families and friends [10–15].

Because peer leadership holds great promise for achieving population health impact, it has been promoted in a variety of settings, including in the United States by the Affordable Care Act [16] and globally by the World Health Organization [17]. However, peer leadership is not without its limitations. Peer leaders often lack prior training in health, pedagogy, and/or other areas central to their role [18]. As such, the scope of work, training, and ongoing support for peer leaders must be appropriate for their role and community context [2, 4, 18, 19]. Currently, little is known about how these roles and contexts shape the experiences of peer leaders, as the vast majority of peer leadership evaluations have focused on intervention recipients rather than on the peer leaders [20–22]. For example, a recent systematic review of peer support programs in cancer care found only 4% of programs focused on program impacts on the peer leaders [21]. Given current efforts to increase the scale of peer leadership models and sustain them in diverse settings, it is critical to understand the experiences of peer leaders and identify the contexts in which they are most successful [18, 19].

### A novel context for peer-led health promotion

Head Start is a federally-funded public preschool program designed to promote the academic development and health of young children in low-income families in the United States [23]. Parent involvement is a central component of Head Start’s efforts to achieve these academic and health outcomes for children [24]; common examples of parent involvement include parenting and health classes taught by Head Start staff [25]. Currently, there are few opportunities in Head Start for parent peer leadership on these topics.

Communities for Healthy Living (CHL), an ongoing childhood obesity prevention trial in Head Start [26] (clinicaltrials.gov NCT03334669), is among the first to engage Head Start parents as peer leaders. Parents are recruited to facilitate a 10-session parent program called Parents Connect for Healthy Living (PConnect). Because many families enroll their children in Head Start for only 1 year [27], high year-to-year turnover is expected among parent PConnect facilitators. Hence, the CHL team decided to pair parent facilitators with staff facilitators, who are more likely to facilitate PConnect for multiple years. This parent-staff co-facilitation model is novel not only because it is being implemented in Head Start, but also because it is focused on the topic of early childhood obesity prevention, an area in which parents are seldom engaged as peer leaders [3, 28]. Given this novelty, it is critical to evaluate the PConnect co-facilitation model, both to inform ongoing efforts to engage parents as parent peer leaders in Head Start and to identify drivers of peer leader success that can apply more broadly.

### Study aim

Peer leader success is multifaceted, with critical early successes including acceptability and appropriateness [29]. For PConnect facilitators, acceptability is defined as satisfaction with being a facilitator and appropriateness is defined as the suitability of being a facilitator (i.e., the role matches their knowledge, skills, etc.) [29]. Simply put, if facilitators do not like their role (low acceptability), or if they do not feel the role is a good fit for them (low appropriateness), it is unlikely that facilitators could be recruited and retained to implement the program. Acceptability and appropriateness are related, yet distinct concepts. Table 1 provides a conceptual model of the relationship between acceptability and appropriateness for intervention implementers. The aim of the current study is to describe the experiences of both staff and parent PConnect facilitators, with a specific focus on why the PConnect co-facilitation model is (not) an acceptable and/or appropriate way to engage parents as peer leaders in the context of Head Start.

**Table 1:** Possible combinations of acceptability and appropriateness for intervention implementers. For PConnect facilitators, acceptability is defined as satisfaction with being a facilitator and appropriateness is defined as the suitability of being a facilitator. Examples are given of how facilitators might describe each combination of acceptability and appropriateness

	Low Acceptability	High Acceptability
**High Appropriateness**	∙ Satisfaction is low ∙ Suitability/fit is high “I don’t like this, but I am capable of doing it.”	∙ Satisfaction is high ∙ Suitability/fit is high “I like this and it is a good fit for me.”
**Low Appropriateness**	∙ Satisfaction is low ∙ Suitability/fit is low “I don’t like this and I don’t have the knowledge, skills, and/or time for it.”	∙ Satisfaction is high ∙ Suitability/fit is low “I like this, but it goes beyond the knowledge, skills, and/or time I have to dedicate to it.”

## Methods

### Study setting and recruitment

CHL is a childhood obesity prevention trial taking place in 16 Head Start programs serving Boston, Cambridge, and Somerville, Massachusetts. Each year, parents and caregivers of enrolled children in the intervention arm of the study are invited to participate in PConnect, a 10-session parent program designed to promote healthy child behaviors and weight through parent empowerment. The initial sessions focus on recommendations for preschoolers’ nutrition, physical activity, sleep, and screen time. The remaining sessions focus on skill-building and connections to resources to help parents achieve these recommendations. PConnect is led by a Head Start parent and a Head Start staff member working together as co-facilitators. All facilitators first complete 12 h of training on the content of the sessions, key group facilitation skills, and strategies for working as an effective co-facilitator pair. The training also teaches facilitators to use the PConnect facilitator guide, which contains a detailed lesson plan for each of the two-hour PConnect sessions. For all of these topics covered by the training, there is first a short lecture-style portion to present the knowledge and skills facilitators will need, followed by activities that allow facilitators to apply that learning and get feedback. These activities include preparing for a PConnect session as a co-facilitator team, and facilitating an activity from that session with the other people in the training playing the role of PConnect participants. Facilitators conduct PConnect sessions in English, Spanish, or Cantonese in their Head Start centers. Further information on PConnect and the CHL intervention can be found elsewhere [26].

**Fig. 1:**
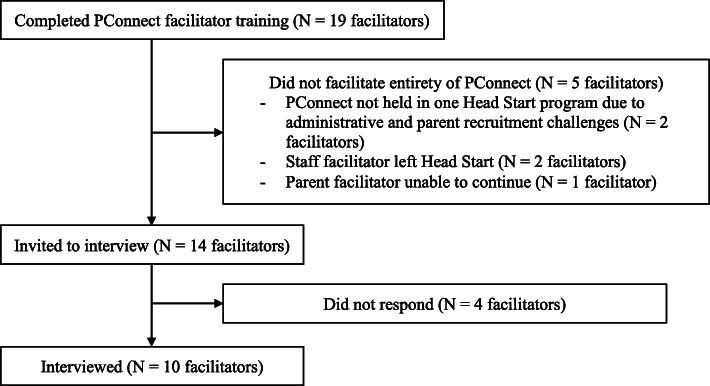
Flow diagram. Of the 19 facilitators who completed the facilitator training, 10 facilitated the entirety of PConnect and participated in a semi-structured interview

In the spring of 2019, nine Head Start programs were scheduled to offer PConnect. The Head Start agencies chose a total of 10 staff members to be staff facilitators, with one program having a back-up staff member to provide support as needed. At each of the nine Head Start programs, program staff recruited one parent facilitator by reaching out directly to parents they knew would be strong candidates for the role. A total of 19 facilitators completed the three-day PConnect facilitator training, 14 of whom implemented PConnect in their Head Start programs. At the conclusion of PConnect, all 14 were invited to participate in a semi-structured interview about their experience. Ten took part in the semi-structured interview in person or over the phone (Fig. 1). CHL staff, who were trained in best practices for qualitative interviews, conducted the 50 min interviews in English, Spanish, or Cantonese based on interviewee preference. Three interviewers had no previous relationship with the facilitators: one female postdoctoral fellow, one female doctoral student, and one male doctoral student. The fourth interviewer, a male doctoral student, led many portions of the facilitator training and knew the facilitators through this work. Interviewees provided consent before participating and received a $20 gift card for their time. The CHL study was approved by the Boston College Institutional Review Board.

The interview guide (Additional file 1) asked questions about the positive and negative aspects of being a facilitator, training and other supports provided, and the impacts of being a facilitator on the facilitators and their families. Questions were developed using the theories that informed the PConnect program, including theories of empowerment (psychological and organizational empowerment [30–32]), as well as theories from implementation science (outcomes for implementation research [29], Consolidated Framework for Implementation Research [33]) and work stress [34].

### Data analysis

Interviews were digitally recorded, transcribed, and translated into English. Interviews were analyzed using an inductive-deductive hybrid thematic analysis [35], which included five steps. First, prior to conducting any analysis, a preliminary codebook was developed based on the theories used to develop the PConnect program and the interview guide [29–34]. Second, JBH and CG analyzed a sample of three transcripts with the preliminary codebook, adding codes inductively to capture phenomena distinct from those in the preliminary codebook. They then met in person, compared coding, and came to consensus on all discrepant coding and inductive codes to be added to the codebook. Third, they independently coded all the interviews; no further inductive codes were found to be needed by either coder. The finalized codebook can be found in Additional file 2. Coded transcripts were compared and all discrepancies were resolved. No discrepancies were found in the underlying meaning attributed to each coded segment; the segments merely had meanings that straddled more than one code. After discussion, all codes deemed appropriate by both coders for each segment were used. Fourth, codes were connected to identify themes. Last, themes were member checked [36] with two facilitators who participated in the interviews and illustrative quotations for each theme were selected. Analytic memos [36] were written and recorded during all stages of analysis. These memos included coders’ thoughts on emerging patterns that became evident during the analytic process; memos were reviewed after all transcripts were coded to aid in the identification of themes. All analysis was conducted in NVivo Version 12 (QSR International Pty Ltd., 2018).

The inductive aspect of the coding process allowed for the possibility of different themes emerging for parent facilitators (conceptualized as peers) and staff facilitators (conceptualized as professionals). This possibility did not materialize; themes applied equally to parent facilitators and staff facilitators. All themes include illustrative quotations from both parent and staff facilitators. To protect the confidentiality of all facilitators, gender-specific pronouns have been replaced with the gender-neutral pronouns “they” and “them” in brackets. The reporting of results follows Standards for Reporting Qualitative Research guidelines [37].

## Results

The facilitators were predominantly female, non-White, and had a college degree or higher (Table 2). Three themes were identified relating to the acceptability of being a PConnect facilitator, and two themes were identified relating to the appropriateness of being a PConnect facilitator. Illustrative quotations are presented below. Supplemental Tables 1–5 in Additional file 3 include interview excerpts illustrating each theme with both the question from the interviewer and the response from the interviewee. Interviewer questions are included to provide greater context and indicate when aspects of the facilitation experience were discussed spontaneously as opposed to being discussed after specific prompting.

**Table 2:** Sample characteristics

	% or Mean (SD) *N* = 10
Female	90%
Age (years)^a^	32.4 (9.6)
Race/ethnicity	
Hispanic/Latino	40%
Non-Hispanic Asian	20%
Non-Hispanic Black/African American	20%
Non-Hispanic White	20%
Education^a^	
High school	10%
Some college	20%
Associate’s or bachelor’s degree	40%
Graduate degree	20%
Facilitator type	
Parent	50%
Staff	50%

^a^Age and education data were missing for one facilitator

### Acceptability theme 1: facilitators are teachers-students

While facilitators were expected to teach and learn given the design of PConnect, an emergent theme from the interviews was that facilitator learning was so prominent that it blurred the traditional line between teachers and students. This concept of simultaneous teaching and learning has been described by Paulo Freire as being a teacher-student [38]. Rather than facilitators assuming the traditional teacher role of disseminating knowledge to participants, who are expected to merely receive that knowledge as students, facilitators and participants alike were working together to learn and improve the health of their children and families.

“It wasn’t just like I was educating them. It was more like I was learning with them, but I was leading the group.” – Staff facilitator 17.

“The first thing that comes to mind [about being a facilitator] would be the knowledge that I gained. I know I’m supposed to be transferring knowledge to the parents, but I gained a lot of knowledge during this program too in working with them and seeing their growth. It’s been great.” – Parent facilitator 13.

### Acceptability theme 2: relationships are valued avenues for the flow of information and emotional support

Facilitators enjoyed fostering relationships in PConnect. Parent facilitators in particular took pride in their ability to develop trust within the group, often by sharing their own experiences. These trusting relationships, in turn, translated to facilitators and participants alike sharing information and emotional support.

“It helps the other parents—comfortable enough that they feel like, ‘Oh, well, [they] went through it. [They’re] doing great. [They’re] able to move on and do things. How can I benefit from that and what [they] has to offer?’” – Parent facilitator 11.

Staff members also valued the relationships they developed with participants, which facilitated the teaching and learning that occurred in PConnect.

“A lot of staff members aren’t able to connect with parents the way that I was able to connect with parents. I think it’s a great opportunity, especially for family advocates who are new to the program. I think it’s great for them to dip their toes in the water and really make those connections and relationships.” – Staff facilitator 23.

### Acceptability theme 3: facilitators are driven by impact

The teaching, learning, and support that facilitators described, which were made possible by the relationships developed, ultimately had powerful impacts on PConnect participants. The sense of making a difference in the lives of PConnect participants was among the most prominent positive aspects of the facilitator experience.

“I like that I’m giving people the skills that they need to make their families healthier and also make their communities healthier, and it’s focusing on empowering the parents. When you empower the parents, you’re empowering the whole family, right?” – Parent facilitator 13.

“It gives that feeling of you’re doing something for the parent ‘cause they’re learning from each other and from what you—information that you’re giving them. It’s so important for the whole family ‘cause they are—not only for them. They give to their families. They make a change in their families.” – Staff facilitator 1.

While facilitators enjoyed having a positive effect on participants, they were frustrated at times by impediments to having an even bigger impact. Low attendance was the most common challenge.

“I think just getting more parents to become involved with the program [was the most challenging part of being a facilitator]. I understand, everybody’s busy with work and stuff like that. Taking 2 h out of the day, that could help benefit you as a better parent and a stronger parent and as a better communicator. I think it’s just very—it’s something that a lot of people should consider. I think definitely just getting more people to wanna be involved in the program.” – Parent facilitator 11.

“The [part I like] least? When they don’t have a good attendance. It’s usually been because of the way some people got jobs, or the kids were sick, or they were sick themselves. […] Sometimes we have things that we have talked about that day. It would be four [participants attending], and then we wanna continue on, and it’s not the same because some of the key people were not there.” – Staff facilitator 1.

Even though most of the discussion around PConnect recruitment and reach focused on the lack of participation as a setback, one facilitator commented on positive aspects of PConnect’s reach.

“I think we did a good job as a team, because it’s not easy. We connect each one with others and we respect the way that everybody approached every topic, and how we achieved the fact that they attended for the 10 weeks.” – Staff facilitator 20.

### Appropriateness theme 1: PConnect provided flexible structure

For the facilitator role to be appropriate, the PConnect program must provide sufficient structure for facilitators to know what to do and how to do it. Facilitators explained that this structure was provided through the PConnect facilitator training and the PConnect facilitator guide, which contains detailed lesson plans for all 10 sessions.

“I think that the training gave us a lot of skills on what to do if things didn’t go the way I’ve planned in the book because the book [PConnect facilitator guide] is pretty straightforward. […] Like I said, it’s well thought out. It’s pretty much everything is there for you in the facilitator guide.” – Parent facilitator 13.

“Someone asked me if they give you training before and I said, ‘Yes, they prepare you for everything that you have to do here.’” – Staff facilitator 20.

A drawback to providing facilitators with highly detailed lesson plans is that it results in a large quantity of materials, which some facilitators found to be overwhelming.

“In the beginning, I felt a little lost with all of the material that we were given. I wasn’t really sure how to navigate it and how to utilize it. It made sense once the program started.” – Staff facilitator 17.

The PConnect training and materials overall provided a clear understanding of what to do in the 10 sessions. When implementing those sessions, facilitators got support from their co-facilitator. Often, co-facilitators had complementary strengths such that when one was having difficulty, the other could help. Common ways that co-facilitators provided this help included support in preparing sessions, leading activities during sessions, and working with participants.

“For me too, it was nice to have someone that I could, I don’t want to say rely on, but just someone I could bounce things off of and, I would ask [them] like, ‘What’s the best way to say this?’ and [they] would help me out with that. That was useful.” – Staff facilitator 25.

“I think [I was least prepared for] maybe some of the questions that some of the parents asked, but it was really great having [name of co-facilitator] there to help me.” – Parent facilitator 11.

While the PConnect facilitator guide provided very detailed lesson plans for each session, the program was not so rigid as to discourage adaptations. Facilitators were specifically asked during the interviews about making changes to the PConnect lesson plans; they explained that they felt comfortable making changes as needed to better suit the needs of participants. Most often, the adaptations made were to the amount of time spent on each activity or to the way activities were completed (e.g., group discussion only rather than writing and discussion).

“Say the first activity went too long, and it was something really important that needed to be addressed […] Then the next activity, I would think, again, this is important, but that was important as well. I know this group of people. I know how I can explain this second activity without going through step by step.” – Staff facilitator 1.

While most facilitators were able to adapt PConnect to participants’ needs, some groups required more adaptation than others. In one case, participants’ deeply held cultural beliefs conflicted with the core content of PConnect activities designed to build personal and political advocacy skills.

“Some of the later sessions I didn’t particularly look forward to or think that they would be helpful for my parents because a lot of them are like advocacy-related or stuff like that. I guess it’s like a cultural thing, but like [name of cultural group] people tend to not really bring their problems to the forefront […] When you mention like, ‘Oh, well, you can talk to this representative, you can bring this up to this person,’ they’re very skeptical about things like that. They’ll say like, ‘Well, that’s not gonna do anything. Even if I say something, nothing’s gonna change.’ That was a little hard.” – Staff facilitator 25.

### Appropriateness theme 2: PConnect facilitation presented manageable challenges

During the interview, facilitators were asked to rate the demandingness of being a facilitator on a scale of 0 to 10. Answers ranged from 3.5 to 9 with an average of 6.3. Most facilitators said that the demands of PConnect had no impact on other areas of their personal or professional lives. Staff facilitators were typically able to balance PConnect with their other responsibilities, and many parent facilitators knew ahead of time that PConnect would not conflict with their schedules.

“Sometimes fitting it into my schedule would be somewhat of a challenge, but it wasn’t extremely challenging ‘cause it’s not that long of a period.” – Staff facilitator 17.

“I actually already spoke with [program coordinator] about being a facilitator back in September, so I knew this was coming and I adjusted my schedule around it.” – Parent facilitator 13.

For the few facilitators who did say PConnect affected other parts of their lives, it was mostly related to finding time to prepare for the sessions. One staff facilitator described needing to use some personal time to prepare for PConnect sessions, which typically took [them] 40 min each week.

“With my family, for example, if I was going to spend a certain amount of time talking to my son, playing, I spent this time reviewing material, because I had to prepare.” – Staff facilitator 20.

One parent facilitator noted feeling “rushed” when managing PConnect with other parts of life, and had trouble finding time to prepare each week. The PConnect facilitator training focused on *how* to use the PConnect facilitator guide to prepare and lead each session, rather than on *what* is in the lesson plan for each session. This parent facilitator suggested spending more time in training reviewing the content of sessions instead.

“I didn’t have time to go through it, sit down and do it ahead of time, but for me, the time to go through it would’ve been during the training, not on a separate day.” – Staff facilitator 14.

A second challenge for some facilitators was finding the right working dynamic with their co-facilitator. Pairs that did experience this issue typically found they were able to resolve it over the course of the first few sessions.

“Nine weeks in, everything is pretty much smooth sailing. In the beginning, it was very hard to get on the same page […] That problem is no longer there. We’re good now, but it was a bumpy start, I would say, in the first two, two and a half weeks.” – Parent facilitator 13.

“I think I did encounter some challenges with my co-facilitator, because I feel like in the beginning they wanted to answer everybody’s question […] We ended up being fine and […] overcoming that.” – Staff facilitator 17.

For other pairs of co-facilitators, it was easy to work together from the beginning.

“My Head Start leader, we worked really well with each other.” – Parent facilitator 14.

“Workin’ with a parent is fantastic. [They] came prepared for every session. We would meet the same day as the session a couple hours before, and [they] would have notes written down, [they] would have asterisks and stars next to what [they] really wanted to talk about. [They’re] very passionate, which made me feel like what we were doin’ was more valuable than I thought we were doing. [They] really enjoyed it so much.” – Staff facilitator 23.

A final common challenge experienced was with language. The study area is very linguistically diverse, so it was not always possible to find facilitators who speak all the languages the parents speak. Even when one facilitator could translate for the other, language was a limitation.

“Another problem was that the—they [PConnect participants] were predominantly speaking in [language removed to protect confidentiality], and I am not a fluent [language removed to protect confidentiality] speaker. That was a problem in the beginning because I felt as though I couldn’t lead as effectively as I wanted to because I don’t have fluency in the language. I had to rely on my co-facilitators to translate.” – Parent facilitator 13.

“It was hard because me myself, I am a native English speaker and I’m also—I’m a native [language removed to protect confidentiality] speaker as well, but I’m not super fluent. I’m more like conversational. When it came to discussing certain things that were more technical, I had a little harder time with that. Fortunately, my co-facilitator was there for that purpose and [they] helped me out with the translational stuff. […] I feel like I could have done this program much better if I had an English speaking group overall, but I think it went well for what it was and for my skill level in terms of the language.” – Staff facilitator 25.

## Discussion

PConnect is among the first programs to engage parents as peer leaders in Head Start, and to do so as part of an early childhood obesity intervention. This novel approach was highly acceptable and appropriate for both the parent facilitators (peer leaders) and the staff facilitators co-leading the program with them; all facilitators expressed interest in facilitating again or said they would recommend being a facilitator to others. The high levels of acceptability were attributable to the fact that PConnect facilitators were able to learn and teach, establish meaningful relationships, and make a positive impact on the parents who participated in their groups. The PConnect co-facilitation approach was appropriate for the parent and staff facilitators because PConnect provided adequate structure and support without being overly rigid, and facilitators were able to overcome the majority of the challenges they encountered.

### Acceptability

There were three major contributors to the high level of acceptability of the co-facilitation model. First, facilitators were not teachers, but teachers-students. Rather than only disseminating knowledge to participants, facilitators also learned from and with participants. While PConnect parent facilitators are not unique among peer leaders in that they learned about health [10–14], it is uncommon that learning from or with program participants is reported to be such a prominent feature of the peer leadership role. Previously, benefits to peer leaders have been framed by the Helper Therapy Principle, which posits impacts on peer leaders are attributable to the personal satisfaction of helping others and to the knowledge and gratitude received from those they help [14, 15, 22]. This phenomenon was certainly apparent in PConnect, but Freire’s concept of teacher-student is more fitting of the way facilitators described the PConnect dynamic because it emphasizes how the traditional power differential between the “helper” and the “helped” was minimized. Shifting existing power structures to empower all involved is at the core of the Freirian approach [39]. Importantly, this teacher-student dynamic was also experienced by staff facilitators. Despite being health and family engagement professionals in Head Start, staff facilitators also described learning from and with participants as a primary benefit of being a facilitator. Beyond PConnect and Head Start, other programs might consider drawing upon Freire’s teacher-student model to enhance the experiences of peer and professional leaders while creating an empowering setting for program participants.

Second, PConnect facilitators took pride in contributing to the relationships that developed in PConnect. Parent facilitators in particular noted that sharing their own experiences helped participants to feel comfortable sharing with one another. This finding is highly concordant with the literature on peer leadership; peers’ ability to establish trust with community members is one of the very reasons for engaging peer leaders in health promotion [8, 9]. Notably, staff facilitators also formed close relationships with PConnect participants. One of the initial motivations for including staff facilitators in PConnect was to create a more sustainable model than a purely parent-led program. An unexpected consequence of this pairing is that it created a unique space for staff members to form relationships with parents. In clinical settings, peer supporters have been integrated into care teams to facilitate patient-provider communication [20]; it is possible that in Head Start, parent facilitators, as peers, similarly aided in communication and therefore relationships between PConnect participants and staff facilitators. Further work is needed to understand when and how peer leaders can help to foster relationships between professionals and community members. It will also be important to investigate how relationships between peer leaders, professionals, and community members may promote intervention effectiveness.

Third, similar to what has been seen among peer leaders elsewhere [10–14, 18, 21, 40], PConnect facilitators found their ability to make an impact on participants highly gratifying. At the same time, as has also been reported among peer leaders elsewhere [18, 40], facilitators were frustrated when they felt their impacts were limited. This finding highlights how appropriateness (suitability) can support acceptability (satisfaction). As Vareilles and colleagues [18] explained, when peer leaders have sufficient skills, knowledge, resources, and support, their role is appropriate. This appropriateness promotes peer leaders’ success in impacting program participants, resulting in a sense of self-efficacy and satisfaction with their role. Across many settings, ensuring the appropriateness of the peer leadership role can support other dimensions of implementation success such as acceptability.

### Appropriateness of PConnect co-facilitation

A first reason that facilitators found their role to be appropriate was that the training and PConnect facilitator guide set clear expectations, and they had support from their co-facilitator in meeting these expectations. Frequently, the staff facilitator and parent facilitator brought complementary strengths. Thus, even if some PConnect facilitator expectations fell outside of one person’s skill set, the co-facilitator pair was able to successfully implement PConnect because that person had support from his/her co-facilitator. In clinical settings, peer leaders have been found to increase the overall capacity of healthcare teams by contributing skills not possessed by clinicians [20]. Our results suggest the partnership between peer leaders and professionals may also be a useful approach in non-clinical settings like Head Start to increase the appropriateness of all people’s roles by allowing them to focus on their strengths.

Another contributor to the appropriateness of the facilitator role was the ability to make adaptations to PConnect. For the PConnect parent facilitator role to be appropriate, parent facilitators need to have the freedom to adjust PConnect sessions according to their deep understanding of their communities’ needs; to prevent parent facilitators from utilizing this important knowledge would reduce the suitability of the role. Adaptability also plays a key role in acceptability; for demanding tasks, a lack of decision latitude results in dissatisfaction [34]. Among health interventions in particular, those with very rigid protocols can be less acceptable [29, 41]. Allowing or even encouraging adaptations, on the other hand, can be empowering for implementers and can improve their sense of involvement in the program, leading to more successful implementation [41]. Finding a balance between fidelity to protocols and adaptation has been identified as a challenge for implementation science broadly [41] and for peer-led programs specifically [42]. For PConnect, the balance targeted was to complete all activities in each session, but allow facilitators to make adaptations to the way in which the activities were implemented. For example, during the PConnect facilitator training, facilitators were encouraged to use their judgment to adjust the amount of time spent on each activity. If a particular activity was proving very productive for their group, they should feel free to extend the amount of time spent on it, and adjust subsequent activities accordingly to finish the session on time. In future studies on facilitation models, it may be important to consider how the balance between fidelity and adaptation impacts the implementation outcomes of appropriateness and acceptability.

A final aspect of the facilitator experience demonstrating its appropriateness is that facilitators were able to overcome common challenges. These challenges included 1) the time commitment required, 2) establishing an effective working relationship between co-facilitators, and 3) overcoming language barriers. All three of these difficulties highlight the importance of peer leader recruitment; peer leaders’ interpersonal skills [19] and language skills [3] have been recruitment criteria used elsewhere to ensure the role is appropriate and that peer leaders can navigate common challenges.

### Implications for peer leadership in Head Start

PConnect is among the first programs in Head Start to incorporate parent peer leadership. The experiences of parent facilitators demonstrate that Head Start parents can be engaged as peer leaders in ways that are both appropriate and acceptable. There is strong potential to implement parent peer leadership more broadly within Head Start because it is highly aligned to Head Start’s two-generation model [24] and to Head Start’s family engagement performance standards (Family partnerships services, 45 CFR § 1302.52) [43]. Specifically, peer leadership may be a way to accomplish two key family outcomes from the Head Start Parent, Family, and Community Engagement Framework: 1) family connections to peers and community, and 2) families as advocates and leaders [44]. Additionally, the co-facilitation model may be a valuable way for Head Start staff members to develop relationships with families. At scale, peer-led health promotion in Head Start has tremendous potential for widespread health impact because Head Start serves nearly three quarters of a million children from low-income families every year [45] and because peer leadership has been found to be a particularly effective approach to health promotion among low-income populations [6]. Further work is needed on other implementation outcomes (e.g., sustainability) and on outcomes for participants (e.g., health knowledge and behavior change) to inform the expansion of peer-led parent programs in Head Start.

### Strengths, limitations, and future directions

A major strength of the current study is that it helps to address the critical need for more work focused on peer leaders’ experiences to inform implementation of peer leadership in diverse settings [20–22]. Moreover, this work was done in Head Start, a novel setting for peer leadership that is well-positioned to engage parents as peer leaders at scale. Another strength of the study is its rigorous methodology, which includes a theoretically-informed interview guide and preliminary codebook, inductive-deductive hybrid thematic analysis, and member checking of themes.

Results must be interpreted with an understanding of key study limitations. One of the four interviewers was involved in the PConnect facilitator training and all were members of the CHL research team, which may have limited the degree to which interviewees shared negative feedback. To address this potential source of bias, the interview guide included prompts specifically about negative aspects of the facilitator experience. Nevertheless, criticisms of PConnect and/or its facilitation model may have been limited. At the analytic stage, acknowledging the potential biases they brought as members of the CHL research team, both coders made a conscious effort to identify negative aspects of the PConnect facilitator experience. Another limitation is that facilitators who did not complete an interview may have had different experiences than those who did. The impact of interview non-response on the study sample size, however, did not present a major limitation; saturation was reached as indicated by the fact that no new inductive codes were added to the codebook by either coder after the first three interviews. A final limitation is that all but one facilitator was a first-time facilitator. The experiences of more experienced facilitators may differ. For example, while the teacher-student dynamic is expected to persist, the amount of new learning facilitators do over time may decrease as they become more familiar with the curriculum.

## Conclusions

Head Start parents and staff alike enjoyed the co-facilitation experience and successfully navigated the challenges entailed in leading a health promotion program in a low-resource setting. This peer leadership model aligns to Head Start’s emphasis on parent engagement, making it a strong candidate for sustained implementation in Head Start. The insights gained about the drivers of appropriateness and acceptability in this particular context may be used to inform the design and implementation of peer-led health programs elsewhere.

## Supplementary Information

**Additional file 1:** Interview Guide.

**Additional file 2:** Codebook.

**Additional file 3: Supplemental Table 1.** Interview excerpts illustrating acceptability theme 1: facilitators are teachers-students. **Supplemental Table 2.** Interview excerpts illustrating acceptability theme 2: relationships are valued avenues for the flow of information and emotional support. **Supplemental Table 3.** Interview excerpts illustrating acceptability theme 3: facilitators are driven by impact. **Supplemental Table 4.** Interview excerpts illustrating appropriateness theme 1: PConnect provided flexible structure. **Supplemental Table 5.** Interview excerpts illustrating appropriateness theme 2: being a facilitator was challenging, but manageable.

## Data Availability

Data can be made available upon request to the corresponding author.

## Abbreviations

CHL: Communities for Healthy Living
PConnect: Parents Connect for Healthy Living
