# Supplementary material for: Acceptability and appropriateness of a novel parent-staff co-leadership model for childhood obesity prevention in Head Start: a qualitative interview study
Source: BMC Public Health. 2021 Jan 22;21:201. doi: 10.1186/s12889-021-10159-3 (PMC7825243; doi:10.1186/s12889-021-10159-3)
Supplement: Supplementary file 1 — Additional file 1: Interview Guide. [file 12889_2021_10159_MOESM1_ESM.docx]

**Additional File 1. Interview guide.**

Introduction for interview:

Thank you for agreeing to speak with me today.  I would like to talk with you about your experience as a PConnect facilitator.  We want to emphasize that this interview is not about evaluating or judging you as a facilitator, or your PConnect group. We will remove your name from the interview transcript and combine your responses with everyone else we interview to learn about PConnect in general. Does this make sense?

There are no right or wrong answers to any of these questions. We just want to learn about your experience as a facilitator so we can improve the program for future PConnect parents and facilitators. Please do not hesitate to share both the good and the bad about your experience. Do you have any questions or concerns before we begin?

1. **How would you describe your experience as a PConnect facilitator?**

Prompt: What is the first thing that comes to mind when you think about your experience as a PConnect facilitator?

1. What did you like most?
2. What did you like least?

1. **Before you started PConnect, you did a 3 day training at the ABCD central office. Now that you have finished facilitating PConnect, what do you think about the training?**
2. Did the training prepare you to be a facilitator? Why/why not?

Prompt:

- What part(s) of being a facilitator were you most prepared for?
- What part(s) of being a facilitator were you least prepared for?
- For Head Start parents and staff in general, do you think the training will prepare them to be a successful facilitator? Why/why not?

1. **Looking back on your experience as a facilitator, what do you think you did well?**

Prompt:

- What are your greatest strengths as a facilitator?
- Are there things you are particularly proud of?

1. **What parts of being a facilitator were most challenging for you? Please remember that we will remove your name from this interview and that this is not about judging you as a facilitator. We want to see if many facilitators encountered similar challenges.**
2. Were you ever worried that one of your sessions would not go well? If so, can you give an example and explain why?

Prompt:

- What do you think were your biggest areas for improvement?
- For facilitators in general, what do you think will be most challenging?

1. **Overall, how was your experience working with your co-facilitator?**
2. What was the best part?
3. The most difficult part? (as needed: remind interviewee about confidentiality and looking for patterns across interviews, not judging individual people)

1. **PConnect parents come from a range of backgrounds/cultures. On a scale of 0 to 10 with 0 being not at all prepared and 10 being completely prepared, how prepared did you feel to work with different types of parents? Why/why not?**
2. Were there any benefits of working with diverse parents? Can you give an example?
3. Were there any challenges? Can you give an example?

1. **Think about all the demands of being a facilitator. This includes time and energy during and between PConnect sessions. On a scale of 0 to 10 with 0 being no demand and 10 being a very high demand, how demanding was it to be a facilitator?**
2. What did you do each week to prepare for sessions?
3. About how much time did you spend preparing each week?
4. Was it difficult to fit this into your schedule? Did it make it harder to find time for work, family, free time, etc?
5. For staff: did PConnect add to your workload, or were you able to do PConnect instead of some other work responsibilities?
6. Did PConnect make it hard for you to get your other work done?
7. Did you ever feel tired or stressed out from being a facilitator?  Did this affect other parts of your life?

1. **You and your co-facilitator met with a coach between sessions to review how things went and to plan for the next session. What did you think of the coaching?**
2. How do you think the facilitator experience would be different without coaching? What would be better? Worse?

Prompt:

i. What was most helpful about the coaching? Can you give an example of how the coaching affected your experience as a facilitator?

ii. What was least helpful? Can you give an example of a time when you didn’t get the support you needed from your coach?

1. **Sometimes changes need to be made to the sessions, like changing the length of an activity, or doing the activity differently than suggested in the facilitator guide. How did you feel about making changes to sessions?**

Prompt:

1. Can you give an example of a change you made and explain why you made it?
2. Were there times that you wanted to change something but you felt you weren’t supposed to?  If so, can you give an example and explain what made you feel that way? Did you end up making the change?

**The next set of questions is about the effects of being a facilitator.**

1. **What impact (good or bad) has being a PConnect facilitator had on you?**
2. **What impact (good or bad) has being a facilitator had on your family? Facilitators with children only: on your children?**

1. **Have you made any changes in what you do personally for your own health because of PConnect?**

Prompt:

- The Healthy Habits (nutrition, physical activity, screen time, sleep); stress management

1. If yes:
2. What changes did you make?
3. Why did you make those changes?

Prompt:

- Learned new information, learned new skills, learned about new supportive resource, got support from others/new social connections, felt more motivated, etc.

1. Do you think you’ll maintain those changes? Why/why not?
2. If no:
3. Was there anything you tried that didn’t work out?
4. Are there any (other) changes you are considering making in the future?

1. **Facilitators with children: Have you made any changes in your parenting because of PConnect? This could be things you do to help your child be healthy or other things you do as a parent.**

Prompt: Healthy Habits, communication strategies, etc.

1. If yes:
2. What changes did you make?
3. Why did you make those changes? (prompt: learned new information, learned new skills, learned about new supportive resource, got support from others/new social connections, felt more motivated, etc.)
4. Do you think you’ll maintain those changes? Why/why not?
5. If no:
6. Was there anything you tried that didn’t work out?
7. Is there anything you do as a parent that you are considering changing in the future?

1. **Does anyone in your family (kids or adults) do anything differently to be healthy because of PConnect?**

Prompt: Healthy Habits, stress management, etc.

1. If yes:
2. What changes did they make?
3. Why did they make those changes? (prompt: a change you made at home, information or skills you taught them, a new resource to help them, support/encouragement you gave them, etc.)
4. Do you think they’ll maintain those changes? Why/why not?
5. If no:
6. Was there anything you encouraged them to do or they tried that didn’t work out?
7. Are there any (other) changes you think they’ll make in the future?

1. **Tell me about your experience working on your Big Goal.**
2. What was your Big Goal?
3. How did you choose it?
4. What did you try? What worked and what didn’t work?
5. What part(s) of PConnect helped you most with your big goal?

Prompt:

- Information, resources, support from other participants, etc.

1. **You already talked about many things you learned as a PConnect facilitator that led you to make changes in what you and your family do to be healthy. Is there anything else you learned in PConnect?**

Prompt:

- Communication strategies (prompt: with your child, with family members/child’s other caretakers, with doctors/teachers/Head Start staff/other professionals, etc.)
- Resources (prompt: at Head Start, in the community, ways to find resources - Neighborhood Resource Map & HelpSteps)
- Ways that you can help friends and others in your community
- Can you give any examples of how your experience as a PConnect facilitator allowed you to help others?

1. **Did you make any friends during PConnect?**
2. Are they parent participants? Your co-facilitator?
3. Do you see these people outside of PConnect now?
4. Do you plan to stay in touch in the future?  How?
5. In what ways do you support or help each other?
6. Did you/do you use WhatsApp/WeChat to keep people in touch with one another?
7. How many people in your PConnect group participated in WhatsApp (or WeChat)?
8. What kinds of things did people talk about or share in your WhatsApp group?

1. **Parent Facilitators Only: Has PConnect made you think about getting more involved in Head Start or in any other community organizations?**

Prompt: For example, during PConnect, did you learn about any important issues/topics in the community? Did you consider getting involved with those issues through Head Start or through some other community organization?

1. If yes: How do you want to get more involved?
2. If no: How come? (note to interviewer: be very careful with tone)

1. **Staff Facilitators Only: Has PConnect made you think about getting more involved in community organizations other than Head Start?**

Prompt: For example, during PConnect, did you learn about any important issues/topics in the community? Did you consider getting involved outside of PConnect?

1. If yes: How do you want to get more involved?
2. If no: How come? (note to interviewer: be very careful with tone)

1. **I asked questions about lots of ways that being a PConnect facilitator may have affected you. Are there benefits I haven’t asked about?**

1. **To make PConnect better in the future, we really want to know about anything negative with being a PConnect facilitator. Is there anything negative about being a PConnect facilitator I haven’t asked about?**

Prompt:

- Time commitment, stress, discussing difficult topics, etc.

1. **Was being a PConnect facilitator worth the time and effort you put into it? Why/why not?**
2. Parent Facilitators only: Would you do it again? Why/why not?

- **What would you say to somebody thinking about becoming a PConnect facilitator?**

1. Would you recommend it? Why/why not?

Theoretical Basis for Interview Questions

| Interview question | Theoretical construct |
| --- | --- |
| 1 | - Acceptability [1] - Appropriateness [1] - Innovation-Values Fit [2] |
| 2 | - Psychological empowerment, emotional[3]/intrapersonal component[4], domain-specific self efficacy - Psychological empowerment, cognitive[3]/interactional component[4], skill development |
| 3 | - Psychological empowerment, emotional[3]/intrapersonal component[4], domain-specific perceived control - Psychological empowerment, emotional[3]/intrapersonal component[4], domain-specific self efficacy - Psychological empowerment, cognitive[3]/interactional component[4], skill development - Psychological empowerment, relational[3] component, facilitating others’ empowerment - Appropriateness [1] |
| 4 | - Psychological empowerment, emotional[3]/intrapersonal component[4], domain-specific perceived control - Psychological empowerment, emotional[3]/intrapersonal component[4], domain-specific self efficacy - Psychological empowerment, cognitive[3]/interactional component[4], skill development - Psychological empowerment, relational[3] component, facilitating others’ empowerment - Appropriateness [1] - Demandingness [5] |
| 5 | - Psychological empowerment, cognitive[3]/interactional component[4], skill development - Psychological empowerment, relational[3] component, collaborative competence - Psychological empowerment, relational[3] component, bridging social divisions - Psychological empowerment, relational[3] component, facilitating others’ empowerment - Appropriateness [1] - Innovation-Values Fit [2] |
| 6 | - Psychological empowerment, emotional[3]/intrapersonal component[4], domain-specific self efficacy - Psychological empowerment, cognitive[3]/interactional component[4], skill development - Psychological empowerment, relational[3] component, collaborative competence - Psychological empowerment, relational[3] component, bridging social divisions - Appropriateness [1] |
| 7 | - Appropriateness [1] - Demandingness [5] |
| 8 | - Innovation-values Fit [2] |
| 9 | - Psychological empowerment, emotional[3]/intrapersonal component[4], domain-specific perceived control - Psychological empowerment, emotional[3]/intrapersonal component[4], domain-specific self efficacy - Psychological empowerment, emotional[3]/intrapersonal component[4], motivation control - Adaptability [2] |
| 10 | - Acceptability [1] - Psychological empowerment, cognitive[3]/interactional component[4], skill development - Psychological empowerment, cognitive[3]/interactional component[4], skill transfer across life domains - Psychological empowerment, cognitive[3]/interactional component[4], resource mobilization |
| 11 | - Acceptability [1] - Psychological empowerment, cognitive[3]/interactional component[4], skill development - Psychological empowerment, cognitive[3]/interactional component[4], resource mobilization |
| 12 | - Psychological empowerment, emotional[3]/intrapersonal component[4], domain-specific perceived control - Psychological empowerment, emotional[3]/intrapersonal component[4], domain-specific self efficacy - Psychological empowerment, emotional[3]/intrapersonal component[4], motivation control - Psychological empowerment, cognitive[3]/interactional component[4], critical awareness - Psychological empowerment, cognitive[3]/interactional component[4], skill development - Psychological empowerment, cognitive[3]/interactional component[4], resource mobilization |
| 13 | - Psychological empowerment, emotional[3]/intrapersonal component[4], domain-specific perceived control - Psychological empowerment, emotional[3]/intrapersonal component[4], domain-specific self efficacy - Psychological empowerment, emotional[3]/intrapersonal component[4], motivation control - Psychological empowerment, cognitive[3]/interactional component[4], critical awareness - Psychological empowerment, cognitive[3]/interactional component[4], skill development - Psychological empowerment, cognitive[3]/interactional component[4], resource mobilization |
| 14 | - Psychological empowerment, emotional[3]/intrapersonal component[4], domain-specific perceived control - Psychological empowerment, emotional[3]/intrapersonal component[4], domain-specific self efficacy - Psychological empowerment, emotional[3]/intrapersonal component[4], motivation control - Psychological empowerment, cognitive[3]/interactional component[4], critical awareness - Psychological empowerment, cognitive[3]/interactional component[4], skill development - Psychological empowerment, cognitive[3]/interactional component[4], resource mobilization - Psychological empowerment, relational[3] component, facilitating others’ empowerment - Psychological empowerment, relational[3] component, network mobilization |
| 15 | - Psychological empowerment, emotional[3]/intrapersonal component[4], domain-specific perceived control - Psychological empowerment, emotional[3]/intrapersonal component[4], domain-specific self efficacy - Psychological empowerment, emotional[3]/intrapersonal component[4], motivation control - Psychological empowerment, cognitive[3]/interactional component[4], critical awareness - Psychological empowerment, cognitive[3]/interactional component[4], skill development - Psychological empowerment, cognitive[3]/interactional component[4], resource mobilization - Psychological empowerment, relational[3] component, network mobilization |
| 16 | - Psychological empowerment, cognitive[3]/interactional component[4], understanding causal agents - Psychological empowerment, cognitive[3]/interactional component[4], skill development - Psychological empowerment, cognitive[3]/interactional component[4], skill transfer across life domains - Psychological empowerment, cognitive[3]/interactional component[4], resource mobilization - Psychological empowerment, relational[3] component, facilitating others’ empowerment - Psychological empowerment, relational[3] component, network mobilization |
| 17 | - Psychological empowerment, cognitive[3]/interactional component[4], resource mobilization - Psychological empowerment, relational[3] component, collaborative competence - Psychological empowerment, relational[3] component, bridging social divisions - Psychological empowerment, relational[3] component, facilitating others’ empowerment - Psychological empowerment, relational[3] component, network mobilization |
| 18 | - Psychological empowerment, emotional[3]/intrapersonal component[4], domain-specific self efficacy - Psychological empowerment, emotional[3]/intrapersonal component[4], motivation control - Psychological empowerment, cognitive[3]/interactional component[4], critical awareness - Psychological empowerment, cognitive[3]/interactional component[4], skill transfer across life domains - Psychological empowerment, relational[3] component, collaborative competence - Psychological empowerment, relational[3] component, network mobilization - Psychological empowerment, behavioral component[4], community involvement |
| 19 | - Psychological empowerment, emotional[3]/intrapersonal component[4], domain-specific self efficacy - Psychological empowerment, emotional[3]/intrapersonal component[4], motivation control - Psychological empowerment, cognitive[3]/interactional component[4], critical awareness - Psychological empowerment, cognitive[3]/interactional component[4], skill transfer across life domains - Psychological empowerment, relational[3] component, collaborative competence - Psychological empowerment, relational[3] component, network mobilization - Psychological empowerment, behavioral component[4], community involvement |
| 20 | - Acceptability [1] - Appropriateness [1] |
| 21 | - Acceptability [1] - Appropriateness [1] - Demandingness [5] |
| 22 | - Acceptability [1] - Appropriateness [1] |
| 23 | - Acceptability [1] - Appropriateness [1] |

References

1. Proctor E, Silmere H, Raghavan R, Hovmand P, Aarons G, Bunger A, et al. Outcomes for implementation research: conceptual distinctions, measurement challenges, and research agenda. Adm Policy Ment Heal Ment Heal Serv Res. 2011;38:65–76.
2. Aarons GA, Palinkas LA. Implementation of Evidence-based Practice in Child Welfare : Service Provider Perspectives. Adm Policy Ment Heal Ment Heal Serv Res. 2007;34:411–9.
3. Christens BD. Toward Relational Empowerment. Am J Community Psychol. 2012;50:114–28.
4. Zimmerman MA. Psychological Empowerment: Issues and Illustrations. Am J Community Psychol. 1995;23:581–99.
5. Karasek RA. Job demands, job decision latitude, and mental strain: implications for job redesign. Adm Sci Q. 1979;24:285–308.
