# Supplementary material for: Acceptability and appropriateness of a novel parent-staff co-leadership model for childhood obesity prevention in Head Start: a qualitative interview study
Source: BMC Public Health. 2021 Jan 22;21:201. doi: 10.1186/s12889-021-10159-3 (PMC7825243; doi:10.1186/s12889-021-10159-3)
Supplement: Supplementary file 2 — Additional file 2: Codebook. [file 12889_2021_10159_MOESM2_ESM.docx]

**Domain 1: Acceptability** – “the perception among implementation stakeholders that a given treatment, service, practice, or innovation is agreeable, palatable, or satisfactory. [… it includes] content, complexity, or comfort”[1]

1. Code name: Overall acceptability
   1. Code description: Facilitators’ overall evaluation of how much they (dis)liked their experience
   2. Code definition: Overall, how much facilitators (dis)liked their experience
      1. Include: Broad, summative evaluations of the facilitator experience, (un)willingness to do it again, (un)willingness to recommend it to others
      2. Exclude: - Reasons why they (dis)liked their experience. More specific codes should be used for reasons why.
   3. Best corresponding interview questions: 1, 10, 11, 22, 23

**Domain 1.1: Psychological empowerment, emotional**[2]**/intrapersonal component**[3] **–** “The intrapersonal component refers to how people think about themselves”[3]

1. Code name: Domain-specific perceived control
   1. Code description: I have the power to control a particular thing. It is within my locus of control.
   2. Code definition: “Domain-specific perceived control refers to beliefs about one's ability to exert influence in different life spheres such as in family, work, or sociopolitical contexts”[3]
      1. Include: Positive/negative feelings about being a PConnect facilitator related to (lack of) control over personal and family health behaviors. This may include proximal factors (setting rules for screen time) or more distal factors (income inequality creates challenges for Head Start families). Also include positive/negative feeling about being a PConnect facilitator related to (lack of) control over the effect of PConnect on participants (e.g., “What I do as a facilitator has the potential to make a big difference in the lives of participants” vs “this type of setting/program can’t make that much of a difference”).
      2. Exclude: Personal ability to control something (this is self-efficacy). Domain-specific perceived control is just whether or not, in theory, a particular thing *could* be controlled (whether or not a specific person has the ability to do it). Also exclude personal desire to control something (this is motivation to control).
   3. Best corresponding interview questions: 3, 4, 9, 12 – 15
2. Code name: Domain-specific self efficacy
   1. Code description: I am confident I have the knowledge/skills/ability to control a particular thing.
   2. Code definition: Having the knowledge/skills/ability to control a particular thing
      1. Include: Positive/negative feeling about being a PConnect facilitator related to (lack of) ability to control personal and family health behaviors. This may include proximal factors (setting rules for screen time) or more distal factors (income inequality creates challenges for Head Start families). Also include positive/negative feeling about being a PConnect facilitator related to (lack of) ability to control the effect of PConnect on participants. This will include strengths and weaknesses as a facilitator only as they are related to the subsequent impact of PConnect on participants. E.g., I am good at X, which created Y benefit for participants. I am not as good at Z, which had a negative effect on participants.
      2. Exclude: General strengths/weaknesses unrelated to outcomes (e.g., Doing X was hard for me, but I got through it and achieved the desired result.).
   3. Best corresponding interview questions: 2a, 3, 4, 6, 9, 12 – 15, 18, 19
3. Code name: Motivation control
   1. Code description: I want to control a particular thing
   2. Code definition: “PE [psychological empowerment] is expected to include a sense of and motivation to control” [3]
      1. Include: Positive/negative feeling about being a PConnect facilitator related to (lack of) desire to control personal and family health behaviors. This may include proximal factors (setting rules for screen time) or more distal factors (income inequality creates challenges for Head Start families). Also include positive/negative feeling about being a PConnect facilitator related to (lack of) desire to control the effect of PConnect on participants. This may be a directly-expressed desire (e.g., I really wanted to…) or one implied by actions if the intended result of those actions is clear (e.g., I spent extra time/effort on…)
      2. Exclude: A lack of action to control something does not imply a lack of desire. A lack of action may relate to perceived control, self-efficacy, or other factors.
   3. Best corresponding interview questions: 9, 12 - 15 (especially the “why” part), 18, 19

**Domain 1.2: Psychological empowerment, cognitive**[2]**/interactional component**[3] **–** “refers to the understanding people have about their community and related sociopolitical issues. This aspect of PE suggests that people are aware of behavioral options or choices to act as they believe appropriate to achieve goals they set for themselves (Zimmerman, 1990a). Individuals must learn about their options in a given context in order to be able to exert control in their environment.”[3]

1. Code name: Critical awareness
   1. Code description: I know what is needed to achieve a goal and how to use those needed things once I have them.
   2. Code definition: “Critical awareness refers to one's understanding of the resources needed to achieve a desired goal, knowledge of how to acquire those resources, and skills for managing resources once they are obtained”[3]
      1. Include: Positive/negative feeling about being a PConnect facilitator related to (lack of) understanding of the knowledge, skills, and/or resources needed to control personal and/or family health behaviors. Also include positive/negative feeling about being a PConnect facilitator related to (lack of) understanding of the knowledge, skills, and/or resources needed to control the effect of PConnect on participants. Especially for parent facilitators, the knowledge/skills/resources required for their own health behavior change may overlap with what is needed by PConnect participants; these things may not be distinguished. However, the knowledge/skills/resources required to be successful in having PConnect participants change health behaviors might also include things related to teaching others effectively and/or motivating others.
      2. Exclude: More distal factors that give rise to health or other issues. These will be coded as “understanding causal agents.” While “critical awareness” is sometimes used to refer to distal/macro/sociopolitical origins of problems, I will define this more as referring to what to do to solve problems (as done by Zimmerman[3]).
   3. Best corresponding interview questions: 12-15, 18, 19
2. Code name: Understanding causal agents
   1. Code description: I understand how current social/political structures/forces impact the people, resources, and events at the root of my (lack of) power.
   2. Code definition: “Understanding causal agents refers to an appreciation of the factors that may influence those people (e.g., mayor, landlord), objects (e.g., toxic chemical, organizational structure), or events (e.g., natural disaster, public hearing) seen to either inhibit or enhance one's efforts to exert control in the sociopolitical environment.”[3]
      1. Include: Positive/negative feeling about being a PConnect facilitator related to (lack of) understanding of social/political structures/forces impacting the people, resources, and events that are important in having power. These are not the immediate causes of issues (e.g., eating too much highly-processed food), but the reasons one may (not) have control over those issues (e.g., I have little power over diet due to long work hours and low minimum wage)
      2. Exclude: Understanding the proximal causes of issues (e.g., sugary drinks cause weight gain). Learning health facts would be coded as “PConnect content.”
   3. Best corresponding interview questions: 16
3. Code name: Skill development
   1. Code description: Building new skills, specifically those relating to decision making, problem solving, and leadership
   2. Code definition: “The interactional component of empowerment also includes decision-making problem-solving, and leadership skills. These skills may be developed in settings where participants have opportunities to become involved in decision making, or inhibited in settings where participation is not an option. It is these skills that help individuals become independent, enable them to control events in their lives, and lead them to become their own best advocates.”[3]
      1. Include: Positive/negative feeling about being a PConnect facilitator related to (lack of) learning how to do something (e.g., using positive guidance to change child behaviors, group facilitation, advocacy)
      2. Exclude: What to do (e.g., recommended health behaviors (this would be “PConnect content”), where to go/who to talk to (this could be “critical awareness” and/or “resource mobilization”)).
   3. Best corresponding interview questions: 2 – 6, 10 – 17
4. Code name: Skill transfer across life domains
   1. Code description: Using skills outside the context of PConnect
   2. Code definition: “A high level of empowerment might be expected among individuals who can generalize skills across life domains, but some individuals may also experience PE in one life domain even if they have been less successful in transferring skills to other life domains.”[3]
      1. Include: Positive/negative feeling about being a PConnect facilitator related to (not) using skills developed through PConnect facilitation outside the realm of PConnect
      2. Exclude: Using skills developed through PConnect facilitation for own/family health behaviors or with PConnect participants (this is not a different life domain)
   3. Best corresponding interview questions: 10, 16, 18, 19
5. Code name: Resource mobilization
   1. Code description: Making use of services, new sources of information, etc. to achieve a goal
   2. Code definition: “Critical awareness refers to one's understanding of the resources needed to achieve a desired goal, knowledge of how to acquire those resources, and skills for managing resources once they are obtained (Kieffer, 1984; Freire, 1973). This ability to mobilize resources is an essential aspect of the interactional component of PE because it suggests environmental mastery.”[3]
      1. Include: Positive/negative feeling about being a PConnect facilitator related to (not) leveraging outside resources. This includes using resources other than those directly provided in PConnect material for personal/family health behavior change (e.g., using a website linked in a PConnect summary sheet, finding a resource independently). This also includes using resources other than those directly provided in PConnect material to impact PConnect participants (e.g., using a website linked in a PConnect summary sheet, finding a resource independently).
      2. Exclude: Mentions of resources provided directly by PConnect. This would be coded as “PConnect resources.” Use of PConnect resources could be considered resource mobilization, but it is a given for facilitators. Use of outside resources captures a perhaps stronger indicator of empowerment.
   3. Best corresponding interview questions: 10 – 17

**Domain 1.3: Psychological empowerment, relational**[2] **component** **–** “A relational component of psychological empowerment can be considered as the psychological aspects of interpersonal transactions and processes that undergird the effective exercise of transformative power in the sociopolitical domain.”[2]

1. Code name: Collaborative competence
   1. Code description: Ability to form a group identity and work together towards a goal
   2. Code definition: “the set of abilities and propensities necessary for the formation of interpersonal relationships that can forge group membership and solidarity […] the ability to act as a part of a group exercising collective agency in the sociopolitical domain […] developing and sustaining successful collaborations and contributing to the development of group solidarity”[2]
      1. Include: Positive/negative feeling about being a PConnect facilitator related to (not) creating friendships/partnerships that provide a sense of group membership/solidarity/community and that act together for a given cause (most likely parent/child/community health in the case of PConnect).
      2. Exclude: Mentions of drawing upon existing relationships/networks to take action on a new or different cause (this would be “network mobilization”). Note that creation of new groups/networks that work on a cause seems to be, by definition, both collaborative competence and network mobilization
   3. Best corresponding interview questions: 5, 6, 17 - 19
2. Code name: Bridging social divisions
   1. Code description: Forming social connections across lines of social status (race, ethnicity, SES, citizenship/immigration status, age, etc.)
   2. Code definition: “Interpersonal activity across diverse settings that develops trust and norms of reciprocity across lines of difference […] propensities and set of competencies necessary for building bridging social capital […]Those who are more adept at bridging social divisions can be expected to understand the roles that isolation and group divisions play in maintaining power asymmetries. Accordingly, they can be expected to possess strategies for bridging social divisions, and to be embedded in relational networks containing others different from themselves.”[2]
      1. Include: Positive/negative feeling about being a PConnect facilitator related to (lack of) friendships/partnerships developed between people of different race/ethnicity, country of birth, religion, culture, age, sex, etc.
      2. Exclude: Friendships/partnership with people in similar situations of similar backgrounds
   3. Best corresponding interview questions: 5, 6, 17
3. Code name: Facilitating others’ empowerment
   1. Code description: Helping others to achieve any dimension(s) of psychological empowerment – this focuses at the level of interpersonal interactions that result in empowerment
   2. Code definition: “the ability and propensity toward facilitating empowering processes for others […] Those who are more empowered in this respect can be expected to demonstrate thoughtfulness and intentionality about group processes, the identification of the capacities of others, and strategies for providing others with key opportunities, supports, and insights.” [2]
      1. Include: Positive/negative feeling about being a PConnect facilitator related to (not) empowering the interviewee’s co-facilitator, participants, family members, friends, etc. (e.g., sharing knowledge, skills, and resources; encouraging leadership)
      2. Exclude: Aspects of interpersonal interactions that do not result in empowerment (e.g., general friendship or social support)
   3. Best corresponding interview questions: 3 - 5, 14, 16, 17
4. Code name: Network mobilization
   1. Code description: Using existing social connections to get people involved and accomplish something
   2. Code definition: “providing a sense of personal invitation to participate […] strengthening commitments to making change on issues that impact those with whom one is in relationship […] motivating and sustaining community mobilization […] mechanisms for the transmission and enactment of cultural and religious influences on collective action […] Those with greater relational empowerment can be expected to be adept at participating both expressively and instrumentally in mobilization processes within the relational networks in which they are participants” [2]
      1. Include: Positive/negative feeling about being a PConnect facilitator related to (not) using relationships to work toward a new goal or take action.
      2. Exclude: Continuing or not changing the actions taken by social networks.
   3. Best corresponding interview questions: 14 - 19
5. Code name: Passing on legacy
   1. Code description: Creating the conditions for others to continue empowering work, relationships, etc. (i.e., for PConnect to continue)
   2. Code definition: “Models and strategies that create and sustain empowering community settings […] involves the commitments of more experienced leaders to investment in the sustainability of their achievements through growth-fostering relationships with those who will succeed them […] not only beneficial for the less experienced members, but can also facilitate growth and development for those with more experience, as well as forging greater group solidarity and capacity”[2]
      1. Include: Positive/negative feeling about being a PConnect facilitator related to (not) taking action taken to help future facilitators and/or the general sustainability of PConnect
      2. Exclude: Sharing knowledge/skills/resources from PConnect with others. This would be “facilitating others’ empowerment.” Passing on the legacy is more specific to continuing the original empowering setting (i.e., PConnect).
   3. Best corresponding interview questions: not specifically assessed

**Domain 1.4: Psychological empowerment, behavioral component**[3] **–** “The behavioral component of PE refers to actions taken to directly influence outcomes.”[3]

1. Code name: Community involvement
   1. Code description: Getting involved in any community group, organization, etc.
   2. Code definition: Influencing outcomes by participating in a community group, organization, etc.
      1. Include: Positive/negative feeling about being a PConnect facilitator related to (lack of) involvement in any community group, organization, etc. that stemmed from being a facilitator (this can include increased involvement in Head Start).
      2. Exclude: Involvement in Head Start that does not extend beyond previous involvement (e.g., existing work responsibilities for staff facilitators, level of parent involvement prior to PConnect).
   3. Best corresponding interview questions: 18, 19

**Domain 1.5: Knowledge**

1. Code name: Health knowledge
   1. Code description: Any mention of health facts or information learned as a result of PConnect
   2. Code definition: Any facts/information about health, including but not limited to physical and mental health for children and adults.
      1. Include: Positive/negative feeling about being a PConnect facilitator related to (lack of) health facts or information acquired from PConnect materials, co-facilitator, and participants.
      2. Exclude: Ways to act on that information. Skills, resources, and behavior change all have their own codes. For example, “children should limit juice” is a health fact that should be coded as health knowledge. “I tried using a smaller cup for juice to limit it” is a way to act on the information that would be coded as health behavior change.
   3. Best corresponding interview questions: 10 – 16
2. Code name: Parenting and family knowledge
   1. Code description: Any mention of facts or information related to parenting or family learned as a result of PConnect
   2. Code definition: Any facts/information about parenting or family.
      1. Include: Positive/negative feeling about being a PConnect facilitator related to (lack of) parenting/family facts and information such as learning about child temperament, parenting styles, communication, cultural differences in family and relationships, etc.
      2. Exclude: Ways to act on that information. Skills, resources, and behavior change all have their own codes.
   3. Best corresponding interview questions: 10 – 16

**Domain 1.6: Behavior change**

1. Code name: Health behavior change
   1. Code description: Changes in health-related behaviors
   2. Code definition: Changes in personal, family (e.g., child, partner, etc.), and/or PConnect participant health-related behaviors
      1. Include: Positive/negative feeling about being a PConnect facilitator related to (lack of) health behavior change. This can include the healthy habits (nutrition, sugary drinks, physical activity, sleep, screen time) and/or other health practices (e.g., working on improved stress management or mental health), This does not have to be a successful or completed change. Include attempted changes and changes in progress.
      2. Exclude: Changes or attempted changes in upstream factors that have the potential to influence health or health behaviors (e.g., communication skills)
   3. Best corresponding interview questions: 10 – 15
2. Code name: Parenting and family behavior change
   1. Code description: Change in approaches to parenting or family relationships
   2. Code definition: Changes in own approaches to parenting/family or PConnect participant approaches to parenting/family
      1. Include: Positive/negative feeling about being a PConnect facilitator related to (lack of) change in parenting or family relationships. Does not have to be a successful or completed change. Include attempted changes and changes in progress. Double-code with health behavior change when the interviewee was involved in a family member making a health behavior change.
      2. Exclude: Changes unrelated to PConnect.
   3. Best corresponding interview questions: 11, 13 – 15

**Domain 1.7: Facilitation stress and challenges**

1. Code name: Facilitation stress and challenges
   1. Code description: Any stress facilitators experience related to their role.
   2. Code definition: Hardship stemming from being a facilitator.
      1. Include: Facilitation challenges that had a negative impact on facilitators’ wellbeing, including direct experiences (e.g., a negative interaction during a PConnect session) and indirect experiences (e.g., feeling overwhelmed by balancing facilitation responsibilities on top of other responsibilities). Also include challenges facilitators say they were able to overcome with no effect on their wellbeing (e.g., learning how to get all participants active in class, which can be challenging but may not negatively impact facilitator wellbeing)
      2. Exclude: n/a
   3. Best corresponding interview questions: 1, 4, 5, 6, **7**, 10, 11, 21 - 23

**Domain 1.8: Relationships**

1. Code name: Friendships
   1. Code description: Any friendships formed or changed through PConnect
   2. Code definition: New friendships and changes to existing friendships resulting from PConnect
      1. Include: Positive/negative feeling about being a PConnect facilitator related to (lack of) friendships formed and changes to existing friendships during PConnect. For existing friendships, can be increased or decreased closeness. There is no lower boundary (i.e., “I wouldn’t call ___ a close friend, but we’re friendly” should be coded here). This can be double-coded with bridging social divisions as appropriate
      2. Exclude: Any people not involved in PConnect. Those relationships may be more appropriately coded with relational empowerment.
   3. Best corresponding interview questions: 17
2. Code name: Social support
   1. Code description: Feeling of being cared for and/or getting help from others
   2. Code definition: Receiving or giving social support, the feeling of being cared for and/or getting help from others
      1. Include: Positive/negative feeling about being a PConnect facilitator related to (lack of) support received from others and support given to others. This includes intangible support (e.g., sense of being cared for) and tangible support (e.g., giving somebody a ride to PConnect).
      2. Exclude: Supports that directly result in empowerment (e.g., teaching somebody how to do something). There will not always be a clear line between supports that are empowering and supports that are not. Double-code when in doubt.
   3. Best corresponding interview questions: 17

**Domain 2: Appropriateness** – “perceived fit, relevance, or compatibility of the innovation or evidence based practice for a given practice setting, provider, or consumer”[1]

1. Code name: Overall appropriateness
   1. Code description: Does the co-facilitation model fit with Head Start staff and parents given their knowledge, skills, job expectations/roles, and other responsibilities?
   2. Code definition: Does the co-facilitation model fit with Head Start staff and parents given their knowledge, skills, job expectations/roles, and other responsibilities?
      1. Include: (Lack of) prior knowledge about the topics covered in PConnect and (lack of) experience with group facilitation or peer leadership, (lack of) ease in integrating PConnect facilitation into personal schedule (e.g., is this too big a time commitment for Head Start parents?), strengths as a PConnect facilitator (suggest sufficient background and/or training to be a successful facilitator), and challenges of being a facilitator (suggest insufficient background and/or training to be a successful facilitator). For staff, also include (lack of) match between PConnect responsibilities and expected job responsibilities, and opportunity to develop professional skills and relationships (note: this can be double coded with skill-building and relational empowerment). For parents, also include opportunity to increase involvement in child’s education (note: this can be double coded with relational empowerment).
      2. Exclude: Appropriateness and acceptability can be strongly related. If something is not appropriate for you, you probably won’t like it (find it acceptable). The converse can be true too; if you really like something, there is a good chance it is appropriate for you. However, appropriateness and acceptability are distinct concepts. Exclude acceptability as much as possible. “Overall appropriateness” is strictly about the fit of being a PConnect facilitator with Head Start parents and staff.
   3. Best corresponding interview questions: 3 – 7
2. Code name: Demandingness
   1. Code description: How difficult it was to be a PConnect facilitator
   2. Code definition: Demand placed on facilitators and how manageable it was in the context of a) the skills, knowledge, experience, etc. facilitators already had and b) other responsibilities that parents and staff manage.
      1. Include: Challenges (or lack thereof) related to being a facilitator. These may be general challenges that may be encountered in any health education setting or challenges specific to the Head Start setting.
      2. Exclude: When possible, exclude (not) liking being a facilitator due to challenges or lack thereof. Otherwise, double-code with acceptability.
   3. Best corresponding interview questions: 4 – 7

**Domain 3: PConnect specifics** – anything related to PConnect training, coaching, content, or implementation

1. Code name: PConnect training
   1. Code description: Did the training match facilitator needs and prepare facilitators to be successful? Did the facilitators (dis)like the training?
   2. Code definition: Did the training prepare facilitators to lead session 1 and lay a strong enough foundation to for facilitators to be successful in the remaining sessions (with the help of the coaching)? Did the training influence how much facilitators (dis)liked their experience as facilitators?
      1. Include: Any aspects of the training that impacted appropriateness of role as a parent or staff facilitator: providing sufficient knowledge of health topics, PConnect materials, PConnect approach (especially relative to background level of knowledge prior to PConnect); providing sufficient skills for group facilitation (especially relative to background level of knowledge prior to PConnect); and logistical difficulties related to training. Also include any difficult aspects of facilitation or times when facilitators felt unprepared (implying potentially inadequate training). Additionally, include positive/negative feeling about being a PConnect facilitator related to training.
      2. Exclude: Mentions of training with no bearing on acceptability or appropriateness.
   3. Best corresponding interview questions: 2 – 4, 6
2. Code name: PConnect coaching
   1. Code description: Did the coaching match facilitator needs and provide the right level of support for facilitators to be successful? Did the facilitators (dis)like the coaching?
   2. Code definition: Did the coaching provide the right level of support for facilitators to be successful? Did the coaching influence how much facilitators (dis)liked their experience as facilitators?
      1. Include: Any aspects of the coaching that impacted appropriateness of role as a parent or staff facilitator: bolstering knowledge and/or skills covered in training, implementing quality improvement to overcome challenges, and logistical difficulties related to coaching. Also include any difficult aspects of facilitation or times when facilitators felt unprepared (implying potentially inadequate coaching) and positive/negative feeling about being a PConnect facilitator related to coaching
      2. Exclude: Mentions of coaching with no bearing on acceptability or appropriateness.
   3. Best corresponding interview questions: 8
3. Code name: PConnect materials
   1. Code description: Did the facilitators (dis)like the facilitation manual, parent binder, etc. Did the materials support facilitator success?
   2. Code definition: Did the facilitators (dis)like the facilitation manual, parent binder, etc. and did the materials support facilitator success?
      1. Include: Positive/negative feeling about being a PConnect facilitator related to the resources provided (acceptability) and degree to which materials support facilitator success (appropriateness). Includes comprehensiveness, ease of use, etc. Note: high degree of overlap between acceptability and appropriateness is likely here. As possible, separate “these resources made me (dis)like being a facilitator” from “these resources made it easy/hard for me to be a successful facilitator.”
      2. Exclude: There may be some overlap between resources and innovation-values fit (e.g., the facilitation manual lays out an activity in a way that does (not) match facilitator values). Double-code these instances. When possible, separate the content of the materials from the materials themselves. It is possible to have a well-designed activity about a topic not very important for parents, or an unclear worksheet about a topic that is important; content and materials are not necessarily the same.
   3. Best corresponding interview questions: 1, 3, 4, 7
4. Code name: PConnect topics
   1. Code description: Did the facilitators (dis)like the topics covered in PConnect? To what extent did the topics covered match the background and comfort level of facilitators?
   2. Code definition: Did the facilitators (dis)like the topics covered in PConnect? Did the facilitators feel they were able to successfully lead sessions on these topics?
      1. Include: Positive/negative feeling about being a PConnect facilitator related to the topics covered in PConnect. This can include finding the topics interesting/useful on a personal level and/or the perception that the topics were interesting/useful for participants. Note: the extent to which PConnect meets the needs of the community is an aspect of PConnect appropriateness more broadly. I am not directly assessing that in this study; this code only includes the extent to which facilitators’ perceptions of PConnect topics impact the acceptability of the co-facilitation model for PConnect specifically (as opposed to PConnect overall). Also include appropriateness considerations: how easy/hard was it for facilitators to lead sessions on these topics, did they have enough knowledge on them, were any topics too sensitive, were any topics too complicated, etc.
      2. Exclude: When possible, separate the topic from the activities and resources associated with it.
   3. Best corresponding interview questions: 1, 3, 4, 12 - 16
5. Code name: Co-facilitation
   1. Code description: Did facilitators (dis)like working with a co-facilitator? Did the partnership with a co-facilitator impact the appropriateness of being a facilitator?
   2. Code definition: Did facilitators (dis)like working with a co-facilitator? Did the partnership with a co-facilitator impact the appropriateness of being a facilitator?
      1. Include: Positive/negative feeling about being a PConnect facilitator related to working with a co-facilitator. This includes (lack of) support provided by the co-facilitator that impacted the appropriateness of the role for the interviewee as well as contributions of knowledge, experience, and/or skills that complement what the interviewee has. Also include (lack of) challenges in coordinating with the co-facilitator to prepare for sessions and facilitate sessions.
      2. Exclude: Anything related to facilitation without explicit mention of the co-facilitator.
   3. Best corresponding interview questions: 5
6. Code name: Innovation-Values Fit
   1. Code description: Does the approach/theory of PConnect match with the values of the facilitators?
   2. Code definition: “‘innovation-values fit’ (Klein & Sorra, 1996) in that implementation may be facilitated when the EBP [evidence-based practice] or innovation fits the provider’s philosophy or approach to provision of services”[4]
      1. Include: Positive/negative feeling about being a PConnect facilitator related to the PConnect approach, including but not limited to: empowerment focus, ecological model, co-facilitation, learning by doing (i.e., each session has at least one activity for participants to apply knowledge and skills covered), and lots of discussion and sharing between participants (not unidirectional flow of knowledge from facilitators to participants).
      2. Exclude: Comments about the specific topics covered or materials used (this would be “PConnect Content”). “Innovation-Values Fit” should focus on how things are done rather than what is done. Note: it is unlikely that this code will be related to appropriateness.
   3. Best corresponding interview questions: 1,5
7. Code name: Adaptability
   1. Code description: Could facilitators adapt PConnect to better match their own strengths and/or the needs of the parents? Or were they forced to work through weaknesses?
   2. Code definition: “The EBP was viewed positively by caseworkers because of its flexibility or adaptability to some specific families and situations. The ability to adapt the delivery, even within the structured intervention was seen as an important positive determinant of implementation.”[4] Adaptability may also related to acceptability through the perspective of the work stress model[5] because it provides insight into control.
      1. Include: Positive/negative feeling about being a PConnect facilitator related to (in)ability to change how it was delivered. Also include any way that the (in)ability to change PConnect delivery impacted facilitators’ success (e.g., (not) being able to change the program to match one’s own strengths).
      2. Exclude: (In)ability to change PConnect related to administrative decisions at the program level (e.g., not having an ideal meeting space)
   3. Best corresponding interview questions: 9
8. Code name: Organizational support
   1. Code description: Did Head Start staff and administrators support facilitators and PConnect in general?
   2. Code definition: Head Start staff and administrator (lack of) support for facilitators and PConnect in general
      1. Include: Positive/negative feeling about being a PConnect facilitator related to meeting space provided, getting help from others at Head Start with recruitment, etc. Also include any way the (lack of) organizational support impacted facilitator success (e.g., ability to balance PConnect with other professional responsibilities). Note: even though CHL staff were responsible for coaching, food ordering, etc., this will eventually be an organizational responsibility if PConnect is continued and it is important to know how this might impact the facilitator experience.
      2. Exclude: Anything related to CHL staff that would not be part of PConnect after the CHL trial (e.g., research consent forms, observations, etc.)
   3. Best corresponding interview questions: 7

References

1. Proctor E, Silmere H, Raghavan R, Hovmand P, Aarons G, Bunger A, et al. Outcomes for implementation research: conceptual distinctions, measurement challenges, and research agenda. Adm Policy Ment Heal Ment Heal Serv Res. 2011;38:65–76.

2. Christens BD. Toward Relational Empowerment. Am J Community Psychol. 2012;50:114–28.

3. Zimmerman MA. Psychological Empowerment: Issues and Illustrations. Am J Community Psychol. 1995;23:581–99.

4. Aarons GA, Palinkas LA. Implementation of Evidence-based Practice in Child Welfare : Service Provider Perspectives. Adm Policy Ment Heal Ment Heal Serv Res. 2007;34:411–9.

5. Karasek RA. Job demands, job decision latitude, and mental strain: implications for job redesign. Adm Sci Q. 1979;24:285–308.
