# Supplementary material for: Acceptability and appropriateness of a novel parent-staff co-leadership model for childhood obesity prevention in Head Start: a qualitative interview study
Source: BMC Public Health. 2021 Jan 22;21:201. doi: 10.1186/s12889-021-10159-3 (PMC7825243; doi:10.1186/s12889-021-10159-3)
Supplement: Supplementary file 3 — Additional file 3: Supplemental Table 1. Interview excerpts illustrating acceptability theme 1: facilitators are teachers-students. Supplemental Table 2. Interview excerpts illustrating acceptability theme 2: relationships are valued avenues for the flow of information and emotional support. Supplemental Table 3. Interview excerpts illustrating acceptability theme 3: facilitators are driven by impact. Supplemental Table 4. Interview excerpts illustrating appropriateness theme 1: PConnect provided flexible structure. Supplemental Table 5. Interview excerpts illustrating appropriateness theme 2: being a facilitator was challenging, but manageable. [file 12889_2021_10159_MOESM3_ESM.docx]

**Supplemental Table 1.** Interview excerpts illustrating acceptability theme 1: facilitators are teachers-students.

| **Acceptability Theme 1: Facilitators are teachers-students** |
| --- |
| Interviewer: What's the first thing that comes to mind when you think about your past experience as a facilitator?  Parent facilitator 13: The first thing that comes to mind would be the knowledge that I gained. I know I'm supposed to be transferring knowledge to the parents, but I gained a lot of knowledge during this program too in working with them and seeing their growth. It's been great. |
| Interviewer: Why would you wanna do it [be a facilitator] again?  Parent facilitator 12: I wanna do it again because I wanna, like I said, learn a little bit more and meet other people, given what I know. |
| Interviewer: The first question I have is how would you describe your experience as a PConnect facilitator?  Staff facilitator 1: I love it. It's really rewarding. […] I just love how the participants learn. I can see how they are learning a lot of stuff. Then they're teaching me as well. I love it. |
| Interviewer: How about [effects] on your family? Your kids, or other adults in your family? Has there been any kind of spillover to them?  Staff facilitator 17: I mean, I definitely learned—I’m a parent myself, so there was things that I learned that I wanna implement in my family. It wasn’t just like I was educating them. It was more like I was learning with them, but I was leading the group, if that makes sense. |
| Interviewer: Do you think it was worth it being a facilitator?  Staff facilitator 20: Absolutely, of course.  Interviewer: Why?  Staff facilitator 20: Because of everything I have told you about how I benefitted. Also I was able to help others, so they could at least understand the steps to take for their children’s development. I was able to share and learn, I think it was very satisfactory. |
| Interviewer: And why [do you say it was worth the effort]?  Staff facilitator 19: Because it gave me the opportunity to share my knowledge and to improve my knowledge. |

**Supplemental Table 2.** Interview excerpts illustrating acceptability theme 2: relationships are valued avenues for the flow of information and emotional support.

| **Acceptability Theme 2: Relationships are the valued avenues for the flow of information and emotional support** |
| --- |
| *Acceptability 2.1: Parent facilitators developed trusting relationships with and among participants* |
| Interviewer: Looking back on your experience as a facilitator, what do you think that you did well? For example, what were your greatest strengths? Are there things that you’re particularly proud of?  Parent facilitator 11: I think the best would be the open communication and where I would be able to influence some of the things that I’ve gone through, as a child, that help other parents open up about similar things that they’ve been through and not being ashamed to talk about very touchy topics, like abuse and neglect and things like that. It helps the other parents—comfortable enough that they feel like, “Oh, well, [they] went through it. [They’re] doing great. [They’re] able to move on and do things. How can I benefit from that and what [they] has to offer?” |
| Interviewer: Do you plan to stay in touch with some of the parents in the future?  Parent facilitator 13: I would hope so, and I would hope that they—if they ever had any questions or anything like that, they would feel comfortable reaching out to me. |
| Interviewer: Moving on from the training now, thinking about just overall your experiences as a facilitator, what do you think you did well?  Parent facilitator 14: I think I did well connection, creating rapport. |
| Interviewer: Did you make any friend at PConnect?  Parent facilitator 16: Making friends? We are all friends now. Aren’t group members our friends? I don’t think that because I am the teacher so I am at a higher position. They are all equal. |

**Supplemental Table 2 (continued).**

| **Acceptability Theme 2: Relationships are the valued avenues for the flow of information and emotional support** |
| --- |
| *Acceptability 2.2: Staff facilitators built relationships with and among parents* |
| Interviewer: Looking back now on your experience as a facilitator, what do you think you did well?  Staff facilitator 17: I think I did well making the parents feel comfortable enough to share whatever they—whatever it is they felt comfortable sharing and creating a space that was safe and nonjudgmental. |
| Interviewer: What would you say was the best part of working with a parent co-facilitator, if you had to choose just one or two things?  Staff facilitator 23: The best part of workin' with my co-facilitator was definitely—we were able to kinda go on a deeper level when it came to our relationship. We were very close before the program. That's why I asked [them] to do the program with me, but [they] opened up about certain topics that [they] wouldn't open up to me if I just said, "Good morning, how are you," in the hallway at school. |
| Interviewer: What would you say to a staff member who asked about being a facilitator?  Staff facilitator 23: I would say it's worth it. […] Also, a lot of people aren't able to—a lot of staff members aren't able to connect with parents the way that I was able to connect with parents. I think it's a great opportunity, especially for family advocates who are new to the program. I think it's great for them to dip their toes in the water and really make those connections and relationships. |
| Interviewer: What do you think were your areas for improvement?  Staff facilitator 19: For me, the area, because I work as the [professional title], was really good because now the parents know me better, and, if they had any question, they know I’m comfortable to answer the question they have. |
| Interviewer: To start, I'd like you to just describe your experience as a PConnect facilitator.  Staff facilitator 25: I enjoyed it. I enjoyed facilitating and I enjoyed developing relationships with the parents at my program. |

**Supplemental Table 3.** Interview excerpts illustrating acceptability theme 3: facilitators are driven by impact.

| **Acceptability Theme 3: Facilitators are driven by impact** |
| --- |
| *Acceptability 3.1: Facilitators are proud of their positive impact on participants* |
| Interviewer: If you had to choose one or two things, what would you say you like most about being a PConnect facilitator?  Parent facilitator 13: I like that I'm giving people the skills that they need to make their families healthier and also make their communities healthier, and it's focusing on empowering the parents. When you empower the parents, you're empowering the whole family, right? |
| Interviewer: How would you describe your experience as a PConnect facilitator?  Parent facilitator 14: Very informative, I really like sharing the information with parents. I like the overall acceptance of the information, and just reaching out to the community was great. |
| Interviewer: Related to just thinking overall about your experience, what would you say you liked most, and what would you say you liked the least?  Staff facilitator 17: What I enjoyed the most was being able to provide families with useful information regarding healthy living. What I liked the least—I don’t really know. |
| Interviewer: Why [did you say you would you recommend being a facilitator to other people]?  Staff facilitator 20. All the benefits you get from sharing information with someone. When you have the opportunity to learn the material in depth to share it with someone, and also receive feedback from someone else, I think it is, uh-huh. You are also contributing to society. You are contributing towards a healthy society. |

**Supplemental Table 3 (Continued).**

| **Acceptability Theme 3: Facilitators are driven by impact** |
| --- |
| *Acceptability 3.1: Facilitators are proud of their positive impact on participants* |
| Interviewer: Okay, and why do you say that [being a PConnect facilitator was worth the time and effort]?  Staff facilitator 23: I think it was worth it because parents were excited about the program, which made me want to really dive into topics. I just wanted to really educate the parents, cuz when it came to some topics they were super-excited, which made me excited to teach. |
| Interviewer: What did you say to her [a prospective facilitator]?  Staff facilitator 1: Yes. I said, yes. You would love it […] It gives that feeling of you're doing something for the parent 'cause they're learning from each other and from what you—information that you're giving them. It's so important for the whole family 'cause they are—not only for them. They give to their families. They make a change in their families |
| Interviewer: Why [did you say you would you be a facilitator again]?  Staff facilitator 19: Because, every year, we have new parents, and all the parents need that great information that this PConnect is givin’ them. |
| Interviewer: To start, I'd like you to just describe your experience as a PConnect facilitator.  Staff facilitator 25: I enjoyed it. […] I think they also enjoyed it as well because they—well, they told me that they looked forward to it every Wednesday. It's nice. I think that through the PConnect program, I was able to provide some education for the parents because they do hear a lot of things on the Internet or through their friends regarding health or nutrition. It was nice to give them information that was not like from—like evidence-based, not just what they can find on the Internet. |

**Supplemental Table 3 (Continued).**

| **Acceptability Theme 3: Facilitators are driven by impact** |
| --- |
| *Acceptability 3.2: Facilitators’ ability to reach parents influenced their sense of impact* |
| Interviewer: What would you say was the most difficult part about working with your co-facilitator?  Parent facilitator 14: […] I guess it didn’t really have to do with [they] or us, but just getting a lot more people involved.  Interviewer: Like recruiting parents?  Parent facilitator 14: Recruitment, yeah, because it really wasn’t on us to do the recruiting. We were relying on a third party to do the recruiting for us, I feel, so I think that was a little bit frustrating for us, ‘cause I feel we were prepared to have more parents than what we did, so I think that was the most difficult part. |
| Interviewer: What parts of being a facilitator were most challenging for you? Remember that we remove your name from the interview. This isn’t about judging you as a facilitator. We just wanna see if many facilitators encountered similar challenges.  Parent facilitator 11: I think just getting more parents to become involved with the program. I understand, everybody’s busy with work and stuff like that. Taking two hours out of the day, that could help benefit you as a better parent and a stronger parent and as a better communicator. I think it’s just very—it’s something that a lot of people should consider. I think definitely just getting more people to wanna be involved in the program. |
| Interviewer: What's the part that you like the least?  Staff facilitator 1: The least?  Interviewer: Yeah.  Staff facilitator 1: Hmm. When they don't have a good attendance. It's usually been because of the way some people got jobs, or the kids were sick, or they were sick themselves. When we have a small group, I—yeah, but then I found out why. Sometimes we have things that we have talked about that day. It would be four [participants attending], and then we wanna continue on, and it's not the same because some of the key people were not there. Then they, well, you have everybody coming too late every session. I know it's not possible all the time. |
| Interviewer: Do you have examples [of things you’re proud of as a facilitator]?  Staff facilitator 20: I think we did a good job as a team, because it’s not easy. We connect each one with others and we respect the way that everybody approached every topic, and how we achieved the fact that they attended for the ten weeks. |

**Supplemental Table 4.** Interview excerpts illustrating appropriateness theme 1: PConnect provided flexible structure.

| **Appropriateness Theme 1: PConnect provided flexible structure to support facilitator success** |
| --- |
| *Appropriateness 1.1: PConnect training and materials provided clear structure* |
| Interviewer: What do you think you were most prepared for coming out of that training?  Parent facilitator 13: Hmm, that's a good question. I think that the training gave us a lot of skills on what to do if things didn't go the way I've planned in the book because the book is pretty straightforward. […] Like I said, it's well thought out. It's pretty much everything is there for you in the facilitator guide. |
| Interviewer: What part, specifically, of being a facilitator were you most prepared for?  Parent facilitator 11: I think knowing how to, basically, take—initiate what was going on and directed in the book and taking that and actually implementing it with the class […] It’s different reading something on paper, but then knowing how to go about it and how to prepare. And like the reading beforehand, you know which articles and materials they need for that session and stuff like that. It just kind of helps you to come more prepared and be really just like cau—not cautious, but more of you know what to expect in a way, rather than just going into it blinded. |
| Interviewer: What comes to your mind when you think about PConnect? What do you like most, and what do you dislike most?  Parent facilitator 16: The best thing I like about PeerConnect is that we have excellent teaching material. It’s very well written by you guys. The material allows facilitators to follow a step by step guide to share with our group members and learn together. |

**Supplemental Table 4 (Continued).**

| **Appropriateness Theme 1: PConnect provided flexible structure to support facilitator success** | |
| --- | --- |
| *Appropriateness 1.1: PConnect training and materials provided clear structure* | |
| Interviewer: What would you say to people that are thinking about becoming a facilitator?  Staff facilitator 20: I would encourage them, I would say ‘You can do it’. Someone asked me if they give you training before and I said, ‘Yes, they prepare you for everything that you have to do here.’ | |
| Parent facilitator 14: I felt like the training was about the flow, and these are all the materials, but if it was possible for each group could’ve gave one different session so that way—just knowing the material a little bit better.  Interviewer: Okay, so actually seeing the sessions in action?  Parent facilitator 14: Seeing the sessions in action and just going through the different sessions. | |
| Interviewer: Now that you have finished facilitating PConnect, what did you think about this training? Do you think it prepared you to be a facilitator?  Staff facilitator 23: I think so. Of course, the sessions aren't gonna run perfectly time-wise, and people getting off topic. You just can't control what people say. It did put into perspective what the goal of the program is, which is great. All the facilitators [staff leading the training] really nailed it on the head when it came to what the purpose of the program was, and what we wanted to give the parents and educate them on. | |
| Interviewer: Now that you've finished facilitating PConnect, what did you think about this training? Do you think it prepared you to be a facilitator or were there parts that you had to learn as you were going along?  Staff facilitator 25: I think both. I did find the training helpful, and it was good that we went through some of the sessions together because that way we could kind of get a feel for how it should run. | |
| Interviewer: Now that you have the experience of doing the whole thing, what are your thoughts about the training?  Staff facilitator 17: The training was good. There was a lot of content that was presented, so I am—it was the three-day training, which was okay. There was a lot of content. I don’t know. In the beginning, I felt a little lost with all of the material that we were given. I wasn’t really sure how to navigate it and how to utilize it. It made sense once the program started. | |

**Supplemental Table 4 (Continued).**

| **Appropriateness Theme 1: PConnect provided flexible structure to support facilitator success** |
| --- |
| *Appropriateness 1.2: Co-facilitators helped in preparing sessions, leading sessions,*  *and working with participants* |
| Interviewer: Were you ever worried that one of your sessions wouldn't go well?  Parent facilitator 13: Not really because I knew that if I was struggling with something, that they [co-facilitators] would be able to help me and pick up where I was lacking. |
| Interviewer: What parts of being a facilitator do you think that you were least prepared for?  Parent facilitator 11: I think maybe some of the questions that some of the parents asked, but it was really great having [name of co-faciltiator] there to help me. |
| Interviewer: You alluded to this a little bit before. You actually said that one thing you thought went really well was working with your co-facilitator. Can you just tell me a little bit more about that? How was your experience working with your Head Start staff member?  Parent facilitator 14: I think that’s an area that [they] was less knowledgeable, and I was more knowledgeable, and we were able to bounce back and forth so that it flowed, and the parents could tell—you know what I mean—that okay—they wouldn’t be able to know the difference. Oh, this one knows less about this subject, or [they] knows more about this subject. When it got to a point where [they] was getting off track, I would jump in, or if timing-wise, if we realized timing, we were just able to bounce off of each other—the flow was really good between us. |

**Supplemental Table 4 (Continued).**

| **Appropriateness Theme 1: PConnect provided flexible structure to support facilitator success** | |
| --- | --- |
| *Appropriateness 1.2: Co-facilitators helped in preparing sessions, leading sessions,*  *and working with participants* | |
| Interviewer: What was a challenge for you in terms of facilitating?  Staff facilitator 23: A big challenge for me was being able to relate on certain topics when it came to mothers and their children, how they would say, "My child does that," or, "Oh, I had the same issue with my child, my son," where I don't have a child, so it makes it hard to be able to also relate to those issues and examples that they were giving out because I don't have a child of my own. I think that's what made it the most difficult, but that's also why I have my co-parent facilitator who is a parent. [They] did help me out in those areas where I lacked in. | |
| Interviewer: What is the best thing about working as a partner?  Staff facilitator 20: The other person can reinforce your weak points. You can switch over. When one is talking, you can check other information, other material, that connection. | |
| Interviewer: What would you say was the best part of working with your co-facilitator?  Staff facilitator 25: […] Learning, I guess, 'cause [they] also took it as like a good opportunity to improve [their] English and to better [their] facilitation skills. For me too, it was nice to have someone that I could, I don't want to say rely on, but just someone I could bounce things off of and, I would ask [them] like, "What's the best way to say this?" and [they] would help me out with that. That was useful. | |
| *Appropriateness 1.3: Adaptability* | |
| Interviewer: It sounds like you definitely were fine moving things around, but you didn't wanna skip anything.  Staff facilitator 1: No, because I'm not a stickler […] Say the first activity went too long, and it was something really important that needed to be addressed […] Then the next activity, I would think, again, this is important, but that was important as well. I know this group of people. I know how I can explain this second activity without going through step by step. | |

**Supplemental Table 4 (Continued).**

| **Appropriateness Theme 1: PConnect provided flexible structure to support facilitator success** |
| --- |
| *Appropriateness 1.3: Adaptability* |
| Interviewer: How did you feel about making those changes when that came up?  Staff facilitator 17: I don’t know. I felt like I—if it, if something needed to be shortened or tweaked, I didn’t really feel any way about it. I just felt like if there was a need to do it, I’m gonna have to do it […] as far as doing it by the book, if I didn’t have time to do it or whatever the case was, then I still had a good feeling about it. I just wanted to make sure that I highlighted it, at least. |
| Interviewer: Can you maybe explain a little more about the changes to session four and maybe why you made those changes?  Staff facilitator 23: Yeah. We finished our session three a little bit earlier, so [co-facilitator’s name] and I, my co-facilitator, were able to talk about how we think it's going so far. We both agreed that we didn't think parents were able to relate on the topic, so we didn't have a set schedule. We made a schedule, we read a schedule every day, or every session on the board, saying what time we were doing this, what time we were doing that. Some parents didn't like it. They would say, "Oh, well, why do you have that? Do we have to go by that?" Once we got used to being able to facilitate, we were able to have more of a relaxed environment when it came to what we were talking about. I think I wouldn't say, "Oh, we're gonna do activity one and two," I'd be like, "Oh, we're gonna look at activity one and two, and we'll choose which activity is best for us to do right now." |
| Interviewer: How did you feel about making changes to those sessions?  Staff facilitator 25: I had no issue with it, particularly because during the training, the people training us, the coaches, they were all very open about that. They told us it's totally fine to change things here and there to fit your group. Not everything's gonna run exactly how it's written in the guide. I felt okay about it overall. |

**Supplemental Table 5.** Interview excerpts illustrating appropriateness theme 2: being a facilitator was challenging, but manageable.

| **Appropriateness Theme 2: Being a facilitator was challenging, but manageable** | |
| --- | --- |
| *Appropriateness 2.1: Time commitment* | |
| Interviewer: What parts of being a facilitator were most challenging for you? I do just wanna remind you that we’re taking your name off the interview, and it’s really not about judging you. We just wanna see if lots of facilitators have similar answers to this type of question.  Parent facilitator 14: […] It’s like for you to explain or be an expert on something, it would be great if I had went through it ahead of time, or just know what it is, and that might be my own fault. I didn’t have time to go through it, sit down and do it ahead of time, but for me, the time to go through it would’ve been during the training not on a separate day. | |
| Interviewer: Did you ever find it difficult to fit this into your schedule, considering that you need—you have time for work and family and free time and those sorts of things?  Parent facilitator 13: Right. I actually already spoke with [program coordinator] about being a facilitator back in September, so I knew this was coming, and I adjusted my schedule around it. | |
| Interviewer: Was it difficult to fit that time, that preparation into your schedule? Did it make it harder to find time for other things, like work, family, free time?  Parent facilitator 11: No, not really. I would try to do it on a day where I didn’t class and when my homework was already done. To take 20 minutes out of my day, it’s really not that bad. | |
| Interviewer: Did you find it difficult to fit this into your schedule? Did you find it hard to find time for work and family and free time when you were having to spend time preparing for the sessions?  Parent facilitator 12: Actually, no. I’m not working at the moment, so it was excellent timing. | |

**Supplemental Table 5 (Continued).**

| **Appropriateness Theme 2: Being a facilitator was challenging, but manageable** |
| --- |
| *Appropriateness 2.1: Time commitment* |
| Interviewer: Did you find it difficult to fit this into your existing schedule? Did it make it harder to find time for your work at Head Start or your family, or did it cut into your free time?    Staff facilitator 25: No, never in my free time, but I think, also for work, I don't really think it affected me too much although I have a large caseload at my program and I do have a lot of things to do, I would find the time and it would work out. It was okay. |
| Interviewer: Did it affect other parts of your life? You said it did.  Staff facilitator 20: With my family, for example, if I was going to spend a certain amount of time talking to my son, playing, I spent this time reviewing material, because I had to prepare. […] I had to reorganize my time. |
| Interviewer: Do you think having to prepare for the sessions made it harder to find time for work, or family, or free time, things like that?  Staff facilitator 23: I mean, I would spend the day before going over all the information, so if I had the session I would teach at—we had Tuesdays and Thursdays, so on a Monday I'd go over it. It wasn't like I was really taking extra time out of my work time. I just had to kind of spread out the two hours of work throughout my actual work day to do this. |
| Interviewer: First question is, how would you describe your experience as a PConnect facilitator?  Staff facilitator 17: I would describe my experience as challenging, but in a good way, and informative. It definitely attributed to my professional development, I think. Sometimes fitting it into my schedule would be somewhat of a challenge, but it wasn’t extremely challenging ‘cause it’s not that long of a period. |

**Supplemental Table 5 (Continued).**

| **Appropriateness Theme 2: Being a facilitator was challenging, but manageable** |
| --- |
| *Appropriateness 2.2: Working with co-facilitator* |
| Interviewer: Is there something that was challenging for you?  Parent facilitator 13: Nine weeks in, everything is pretty much smooth sailing. In the beginning, it was very hard to get on the same page as my co-facilitators […] That problem is no longer there. We're good now, but it was a bumpy start, I would say, in the first two, two and a half weeks. |
| Interviewer: Thinking about just overall your experiences as a facilitator, what do you think you did well?  Parent facilitator 14: […] my Head Start leader, we worked really well with each other. |
| Interviewer: Overall, how was your experience working with your co-facilitator, the staff member at Head Start?  Parent facilitator 12: It was excellent. I really enjoyed it. |
| Interviewer: How about what parts of being a facilitator were most challenging for you?  Staff facilitator 1: […] I did it with a new one [co-facilitator], but it still—we did good. It was a challenge in the beginning. |
| Interviewer: How about, what parts of being a facilitator were most challenging for you? Again, it’s really, it’s not about judging you specifically in like, oh, you didn’t do this well. It’s really more to see if maybe lots of facilitators encountered similar challenges.  Staff facilitator 17: Mm-hmm. In the beginning, it was a little bit of a challenge to, not necessarily—it wasn’t a challenge as a whole, but we did, I think I did encounter some challenges with my co-facilitator, because I feel like in the beginning they wanted to answer everybody’s question. I felt like I didn’t wanna give information that wasn’t true, but at the same—in the same way, I didn’t know how to address that without being offensive. We ended up being fine and—what am, what’s the word I’m looking for? Overcoming that. Yeah. Maybe just, in the beginning, setting some standards between the co-facilitator—the parent co-facilitator and the head start facilitator, as to what the expectations are as far as, I don’t know, just leading the group, I guess. |

**Supplemental Table 5 (Continued).**

| **Appropriateness Theme 2: Being a facilitator was challenging, but manageable** |
| --- |
| *Appropriateness 2.2: Working with co-facilitator* |
| Interviewer: How did you eventually connect with each other?  Staff facilitator 20: I think when we go deep in the material that you gave to us, that material explain how to do it, how are we going to talk, what’s easier, because everybody knows what to talk , everybody knows what to do, that’s it. |
| Interviewer: Overall, how would you characterize this experience, working with the parent?  Staff facilitator 23: Workin' with a parent is fantastic. [They] came prepared for every session. We would meet the same day as the session a couple hours before, and [they] would have notes written down, [they] would have asterisks and stars next to what [they] really wanted to talk about. [They’re] very passionate, which made me feel like what we were doin' was more valuable than I thought we were doing. [They] really enjoyed it so much. |
| *Appropriateness 2.3: Language barriers* |
| Interviewer: Is there something that was challenging for you?  Parent facilitator 13: […] Another problem was that the—they were predominantly speaking in [language removed to protect confidentiality], and I am not a fluent [language removed to protect confidentiality] speaker. That was a problem in the beginning because I felt as though I couldn't lead as effectively as I wanted to because I don't have fluency in the language. I had to rely on my co-facilitators to translate. |
| Interviewer: How prepared did you feel to work with different types of parents?  Parent facilitator 14: I think that we were very prepared to work with different types of parents. We did have some people where they did not speak—their first language wasn’t English […] even though I don’t speak [language removed to protect confidentiality], it was good because we were able to explain a little bit better, or slow down when there was a language barrier. |

**Supplemental Table 5 (Continued).**

| **Appropriateness Theme 2: Being a facilitator was challenging, but manageable** |
| --- |
| *Appropriateness 2.3: Language barriers* |
| Interviewer: On scale of zero to 10, with zero being not at all prepared and 10 being completely prepared, how prepared did you feel to work with different types of parents? Parents from different backgrounds or cultures or genders.  Parent facilitator 12: I’d say about a seven. Most parents speak in different language and didn’t quite understand, and I’m not bilingual. […] I showed them pictures. Then I had the other facilitator. [They] was talking in their language. |
| Interviewer: What is the first thing that comes to mind when you think about your experience being a PConnect facilitator?  Staff facilitator 25: I think of like the discussions that we've had […] it was hard because me myself, I am a native English speaker and I'm also—I'm a native [language removed to protect confidentiality] speaker as well, but I'm not super fluent. I'm more like conversational. When it came to discussing certain things that were more technical, I had a little harder time with that. Fortunately, my co-facilitator was there for that purpose and [they] helped me out with the translational stuff. […] I feel like I could have done this program much better if I had an English speaking group overall, but I think it went well for what it was and for my skill level in terms of the language. |
